# In planta expression of human polyQ-expanded huntingtin fragment reveals mechanisms to prevent disease-related protein aggregation

**DOI:** 10.1038/s43587-023-00502-1

**Published:** 2023-10-02

**Authors:** Ernesto Llamas, Seda Koyuncu, Hyun Ju Lee, Markus Wehrmann, Ricardo Gutierrez-Garcia, Nick Dunken, Nyasha Charura, Salvador Torres-Montilla, Elena Schlimgen, Amrei M. Mandel, Erik Boelen Theile, Jan Grossbach, Prerana Wagle, Jan-Wilm Lackmann, Bernhard Schermer, Thomas Benzing, Andreas Beyer, Pablo Pulido, Manuel Rodriguez-Concepcion, Alga Zuccaro, David Vilchez

**Affiliations:** 1 Cologne Excellence Cluster for Cellular Stress Responses in Aging-Associated Diseases, University of Cologne, Cologne, Germany; 2 Cluster of Excellence on Plant Sciences, Institute for Plant Sciences, University of Cologne, Cologne, Germany; 3 Institute for Integrated Stress Response Signaling, Faculty of Medicine, University Hospital Cologne, Cologne, Germany; 4 Institute for Plant Molecular and Cell Biology CSIC-UPV, Valencia, Spain; 5 Department II of Internal Medicine, University Hospital Cologne, Cologne, Germany; 6 Institute for Genetics, University of Cologne, Cologne, Germany; 7 Center for Molecular Medicine Cologne, University of Cologne, Cologne, Germany; 8 Department of Plant Molecular Genetics, Centro Nacional de Biotecnologia, Consejo Superior de Investigaciones Científicas, Madrid, Spain

**Keywords:** Biological techniques, Physiology, Mechanisms of disease, Ageing

## Abstract

In humans, aggregation of polyglutamine repeat (polyQ) proteins causes disorders such as Huntington’s disease. Although plants express hundreds of polyQ-containing proteins, no pathologies arising from polyQ aggregation have been reported. To investigate this phenomenon, we expressed an aggregation-prone fragment of human huntingtin (HTT) with an expanded polyQ stretch (Q69) in *Arabidopsis thaliana* plants. In contrast to animal models, we find that *Arabidopsis* sp. suppresses Q69 aggregation through chloroplast proteostasis. Inhibition of chloroplast proteostasis diminishes the capacity of plants to prevent cytosolic Q69 aggregation. Moreover, endogenous polyQ-containing proteins also aggregate on chloroplast dysfunction. We find that Q69 interacts with the chloroplast stromal processing peptidase (SPP). Synthetic *Arabidopsis* SPP prevents polyQ-expanded HTT aggregation in human cells. Likewise, ectopic SPP expression in *Caenorhabditis elegans* reduces neuronal Q67 aggregation and subsequent neurotoxicity. Our findings suggest that synthetic plant proteins, such as SPP, hold therapeutic potential for polyQ disorders and other age-related diseases involving protein aggregation.

## Abstract (web summary)

Unlike animals, plants prevent pathological polyQ aggregation through chloroplast proteostasis. Expression of the chloroplast protein SSP in human cell and nematode models prevents disease-related protein aggregation and neurodegeneration.

## Main

Across the proteome, numerous proteins are prone to self-assembly into pathological aggregates^1^. Many human neurodegenerative diseases involve proteins with prion-like domains or intrinsically disordered regions rich in asparagine (N) and glutamine (Q) residues, which promote aggregation^2^. For instance, a common feature of polyQ-containing proteins is their capacity to form aggregates in yeast and higher eukaryotes^3^. However, cells have evolved proteostasis mechanisms to prevent the harmful aggregation of polyQ-expanded proteins, including degradation through the ubiquitin–proteasome system and disaggregation by chaperones^4–10^.

At least nine human neurodegenerative diseases are associated with polyQ-containing proteins. Among them, Huntington’s disease is caused by mutations in exon 1 of the huntingtin (*HTT*) gene that expands the polyQ stretch of the protein^11,12^. The wild-type HTT protein contains 6–35 polyQ repeats^13,14^ and does not aggregate even under stress conditions or during aging^9^. In individuals affected by Huntington’s disease, an unstable expanded polyQ stretch (>Q35) causes aggregation and proteotoxicity. The pathogenic fragment of polyQ-expanded exon 1 of mutant HTT in different model organisms and human cells is sufficient to recapitulate key aspects of Huntington’s disease, including pathological protein aggregation and cell death^15–17^. Another protein associated with a human disease is ATXN3, which can contain up to 52 polyQ repeats without forming aggregates, even under challenging conditions^9^. However, a mutant polyQ extension beyond 52 repeats triggers ATXN3 aggregation, causing Machado–Joseph disease^18,19^.

Although plants express hundreds of proteins containing polyQ regions^20^, no pathologies arising from these proteins have been reported to date. In contrast to human HTT and ATXN3, which have relatively long polyQ repeats in their wild-type forms, the polyQ stretch in the *A. thaliana* proteome does not exceed 24 repeats^20^. It is interesting that specific polyQ proteins act as sensors that integrate internal and external cues, enabling *Arabidopsis* sp. to adapt to its ever-changing environment^21–23^. One example is the transcription factor EARLY FLOWERING 3 (ELF3), which contains a Q7 stretch that allows the plant to respond to high temperatures through its aggregation. At 22 °C, ELF3 remains soluble and binds to genes that repress flowering. At temperatures higher than 27 °C, ELF3 forms aggregates that relieve transcriptional repression and promote flowering^21,24^. Thus, ELF3 can form aggregates in *Arabidopsis* sp. under stress conditions, even with its relatively short Q7 motif^21^.

As the longest polyQ expansion in *Arabidopsis* proteins is 24 repeats^20^, we expressed the exon 1 of human HTT containing Q28 and Q69 to examine whether plants can cope with polyQ-expanded proteins. Under normal conditions, neither Q28 nor Q69 leads to the formation of aggregates or deleterious effects in *Arabidopsis* sp. However, similar to *Arabidopsis* ELF3 (Q7)^21^, both Q28 and Q69 accumulate into aggregates on heat stress. Under nonstress conditions, *Arabidopsis* sp. efficiently prevents aggregation of polyQ-expanded proteins through their import and degradation into the chloroplast. Conversely, disruption of chloroplast proteostasis either pharmacologically or genetically triggers the cytosolic aggregation of Q69 as well as endogenous polyQ proteins. We found that both Q28 and Q69 interact with various chloroplast proteins, such as the SPP. Notably, ectopic expression of SPP reduces the aggregation of polyQ-expanded proteins in human cells and nematode models. These findings open up an avenue for the discovery of therapeutic, plant-based synthetic proteins that could target human polyQ diseases.

## Results

### *Arabidopsis* sp. prevents Q69 aggregation under normal conditions

**Fig. 1:**
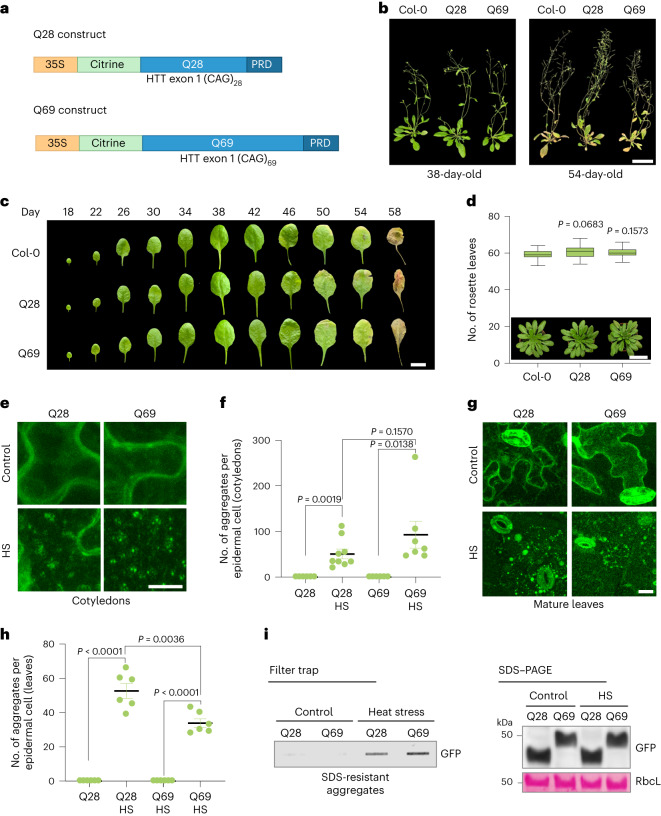
Plants constitutively expressing polyQ-expanded proteins do not display aggregates or deleterious effects under normal conditions. **a**, Schematic representation of the constitutive constructs Q28 and Q69. PRD, proline-rich domain. **b**, Phenotype of mature and senescent wild-type Col-0, Q28 and Q69 plants. Scale bar, 5 cm. The results represent three independent experiments. **c**, Lifespan analysis of the fourth true leaf of *Arabidopsis* sp. comparing Q28 and Q69 with control Col-0 plants. Scale bar,1 cm. The results represent three independent experiments. **d**, Representative images of 46-day-old plants. Scale bar, 5 cm. The box plot represents the 25th–75th percentiles of the flowering time under short-day conditions at 22 °C (*n* = 27 biological replicates), the line depicts the median and the whiskers are plotted following Tukey’s method. **e**, Confocal images of citrine-Q28 and citrine-Q69 (citrine is a fluorescent protein derived from GFP) in epidermal pavement cells from cotyledons. The 7-day-old seedlings grown at 22 °C were transferred in the dark to incubators at 45 °C (heat stress (HS)) or 22 °C (control) for 90 min. Scale bar, 10 μm. The results represent three independent experiments. **f**, Quantification of the number of Q28 and Q69 aggregates per epidermal cell in 7-day-old cotyledons from the experiments presented in **e** (mean ± s.e.m.; Q28, *n* = 6 cells from three independent experiments; Q28 HS, *n* = 9; Q69, *n* = 6; Q69 HS, *n* = 7). **g**, Q28 and Q69 distribution in epidermal pavement cells from leaves of 22-day-old plants. The fourth leaf of Q28 or Q69 plants was dissected and incubated in the dark under HS or control conditions for 90 min. Scale bar, 25 μm. The results represent three independent experiments. **h**, Quantification of Q28 and Q69 aggregates per epidermal cell in 22-day-old leaves from **g** (mean ± s.e.m.; *n* = 6 cells per condition from three independent experiments). **i**, Filter trap and SDS–PAGE analysis with anti-GFP antibody (capable of recognizing citrine tag) of the seedlings used for microscopy analysis in **e**. Rubisco large subunit (RbcL) is the loading control. The results represent two independent experiments. Statistical comparisons were made using two-tailed Student’s *t*-test for unpaired samples. Source data

**Extended Data Fig. 1:**
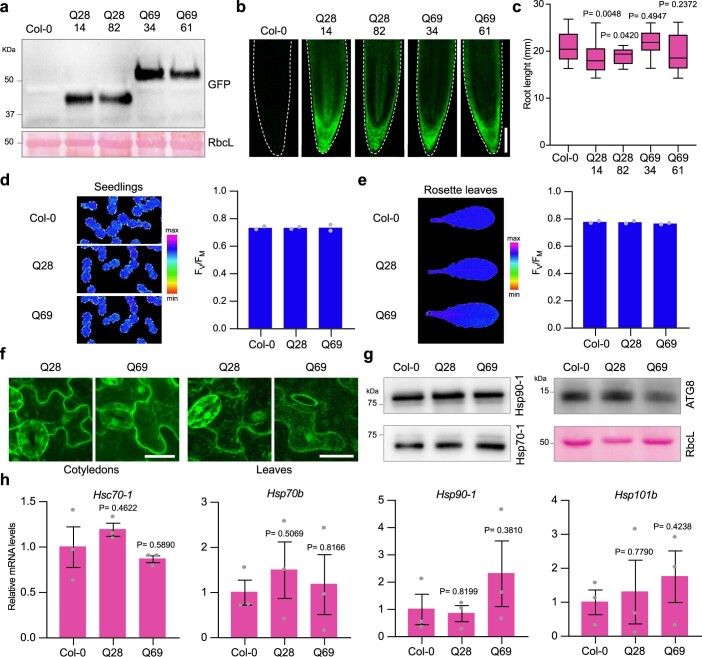
Characterization of plants constitutively expressing Q28 and Q69. **a**, Immunoblot analysis with anti-GFP antibody of Col-0 wild-type controls and independent transgenic Citrine-Q28 and Citrine-Q69 plants. Citrine is a fluorescent protein derived from GFP. Ponceau S staining showing RbcL is the loading control. Representative of two independent experiments. **b**, Microscopy analysis of 7-day-old Arabidopsis transgenic plants expressing Q28 and Q69. Scale bar: 100 μm. Representative of three independent experiments. **c**, Root growth analysis of 7-day-old Col-0, Q28 and Q69 seedlings (n = 30 roots). The box plot represents the 25th–75th percentiles, the line depicts the median and the whiskers are plotted following the Tukey method. Statistical comparisons were made by ordinary one-way ANOVA. **d-e**, Photosynthetic activity F_V_/F_M_ of 7-day-old Col-0, Q28 and Q69 seedlings (**d**) and rosette leaves of 38-day-old plants (**e**) measured via PAM fluorometry. Data from two independent measurements. **f**, Confocal microscopy analysis of Q28 and Q69 in cotyledons of 7-day-old seedlings and the fourth true rosette leaves of 22-day-old Arabidopsis plants. Scale bar: 20 μm. Representative of four independent experiments. **g**, Immunoblot analysis of proteostasis marker proteins in 7-day-old seedlings. RbcL is the loading control. Representative of three independent experiments. **h**, Real-time quantitative PCR (qPCR) analysis of 7-day-old-seedlings grown under normal conditions (22 °C). Proteostasis-related genes are not upregulated in polyQ-expressing lines compared to control Col-0. Graph (relative expression to Col-0) represents the mean ± s.e.m. of three independent experiments. Statistical comparisons were made by two-tailed Student’s *t*-test for unpaired samples. Source data

In invertebrate and mammalian model organisms, the expression of HTT exon 1 containing >35 glutamine repeats is sufficient to trigger polyQ aggregation^6,25,26^. To recapitulate the pathological aggregation phenotype of Huntington’s disease in plants, we generated transgenic *Arabidopsis* sp. expressing the human mutant HTT exon 1 fragment. To this end, we generated the constructs *35S:Citrine-HTTexon1-Q28* (Q28) and *35S:Citrine-HTTexon1-Q69* (Q69) (Fig. 1a). Subsequently, we established and characterized *Arabidopsis* transgenic plants expressing Q28 and Q69 under the control of the 35S promoter (Fig. 1b–d and Extended Data Fig. 1a–g). Constitutive expression of Q28 and Q69 did not cause deleterious effects in *Arabidopsis* plants, which exhibited similar development, lifespan, flowering time and photosynthetic activity compared with untransformed, Col-0 wild-type controls (Fig. 1b–d and Extended Data Fig. 1c–e).

**Extended Data Fig. 2:**
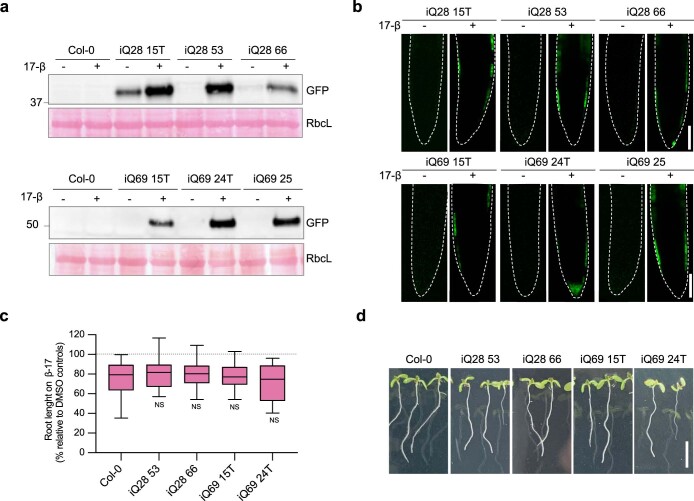
Characterization of inducible Q28 (iQ28) and Q69 (iQ69) plants. **a**, Immunoblot analysis against GFP of independent transgenic iQ28 and iQ69 plants. Ponceau S staining showing RbcL is the loading control. Representative of two independent experiments. **b**, Microscopy analysis of 7-day-old Col-0, iQ28 and iQ69 seedlings germinated and grown on vertical plates supplemented with 10 μM 17-β-estradiol (17-β). Scales bar: 100 μm. Representative of three independent experiments. **c**, Root length of plants grown on plates supplemented with 10 μM 17-β-estradiol relative to their corresponding controls grown on DMSO vehicle control. Statistical comparisons between Col-0 and polyQ plants were made by two-tailed Student’s t test for unpaired samples (Col-0 *n* = 20; iQ28 53 *n* = 12; iQ28 66 *n* = 13; iQ69 15 T *n* = 18; iQ69 24 T *n* = 20). NS= not significant (*P* > 0.05). The box plot represents the 25th–75th percentiles, the line depicts the median and the whiskers are plotted following the Tukey method. **d**, Images of 7-day-old Col-0, iQ28 and iQ69 plants treated with 10 μM 17-β estradiol. Scale bar: 5 mm. Representative of two independent experiments. Source data

We observed a diffuse distribution pattern for both Q28 and Q69 proteins in the root tips, cotyledons and mature leaves of plants under normal growth conditions (Extended Data Fig. 1b,f). Moreover, polyQ-expanded proteins did not induce proteostasis stress markers, indicating the absence of proteotoxicity in these transgenic lines (Extended Data Fig. 1g,h). To tightly control the expression of polyQ proteins, we generated inducible transgenic plants that express Q28 or Q69 in the presence of estradiol. After 7 d of estradiol treatment, we did not observe aggregation or toxic effects in either inducible Q28 or Q69 seedlings (Extended Data Fig. 2a–d). Together, our results indicate that *Arabidopsis* plants have mechanisms to sustain proteostasis and prevent polyQ aggregation throughout their life.

**Extended Data Fig. 3:**
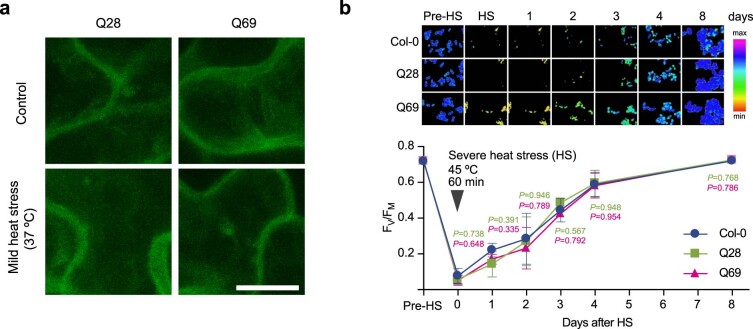
PolyQ28 and Q69-expressing plants do not exhibit increased sensitivity to heat stress compared to wild-type plants. **a**, Images of epidermal cells from cotyledons. 7-day-old seedlings grown at 22 °C were transferred in dark to incubators at 37 °C (mild heat stress) or 22 °C (control) for 90 minutes. Scale bar: 10 μm. In contrast to severe heat stress (45 °C, please see Fig. 1e–i), mild heat stress (37 °C) does not cause polyQ aggregation. Representative of three independent experiments. **b**, Control wild-type Col-0, Q28 and Q69 7-day-old-plants grown at 22 °C in short-day (SD) conditions were transferred to 45 °C for 60 minutes. After severe heat shock (HS), the plants were transferred back to 22 °C (SD). Photosynthetic parameters were measured before HS, immediately after HS, and at day 1, 2, 3, 4, and 8 after HS. Upper panel shows the visualization of F_V_/F_M_ measured via PAM fluorometry. Blue indicates a high photosynthetic activity. Green and yellow indicates decreased and strongly decreased photosynthetic activity, respectively. Graph represents the mean ± s.e.m of 3 independent experiments. Statistical comparisons were made by two-tailed Student’s *t*-test for unpaired samples. Source data

In humans, HTT and ATXN3 can contain up to 35 and 52 polyQ repeats, respectively, before becoming prone to aggregation even under stress conditions^9,12,18^. In contrast, the polyQ stretches in endogenous *Arabidopsis* proteins do not exceed 24 glutamine repeats^20^ (Supplementary Table 1). Among them, ELF3 protein can form aggregates at higher temperatures even with a short poly(Q7) stretch^21^. We hypothesized that, unlike animals^26,27^, relatively shorter polyQ stretches are prone to aggregation in plants during stress conditions. Thus, plants might require intrinsic proteostasis mechanisms to avoid polyQ aggregation under normal conditions. To assess whether elevated temperatures trigger polyQ-expanded aggregation, we subjected 7-day-old stable transgenic plants expressing Q28 and Q69 to either mild (37 °C) or severe (45 °C) heat stress for 90 min. Although mild stress conditions did not cause aggregation of cytosolic Q28 and Q69 (Extended Data Fig. 3a), a severe heat stress led to the formation of Q28 and Q69 aggregates (Fig. 1e–i). However, Q28 and Q69 seedlings did not exhibit increased sensitivity to heat stress compared with wild-type plants (Extended Data Fig. 3b).

### Q69 interacts with chloroplast proteostasis components

**Fig. 2:**
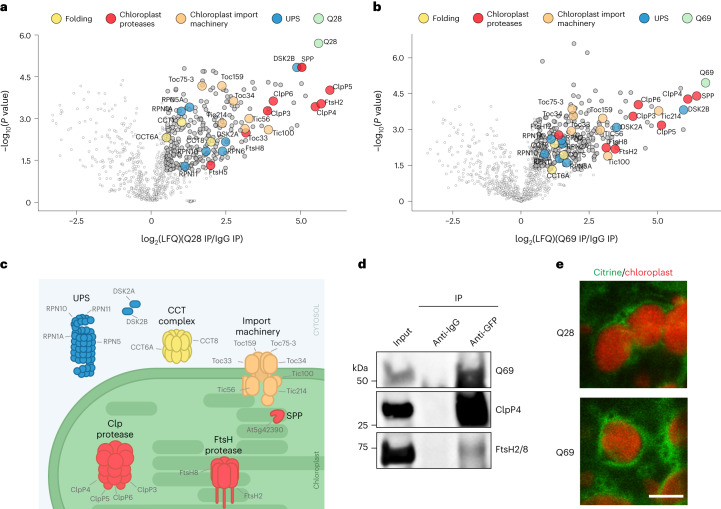
Q28 and Q69 proteins interact with cytosolic and chloroplast proteostasis components in *Arabidopsis* sp. **a**,**b**, Co-immunoprecipitation (co-IP) experiments with anti-GFP antibody against *Citrine-HTTexon1-Q28* (**a**) and *Citrine-HTTexon1-Q69* (**b**) in transgenic 7-day-old *Arabidopsis* seedlings, followed by quantitative label-free proteomics. For each biological replicate, the same amount of protein was incubated with either anti-GFP or negative control anti-IgG antibody. To identify significant interactors of Q28 and Q69, we compared protein abundance in GFP pulldowns with control IgG pulldowns. Volcano plots represent the −log_10_(*P* value) of a two-tailed Student’s *t*-test plotted against the log_2_(ratio) of protein LFQ values from GFP pulldown compared with control IgG pulldown (Student’s *t*-test, *n* = 3 biological replicates). Gray and colored circles indicate the significance after correction for multiple testing (FDR < 0.05 was considered significant). Yellow circles indicate proteins involved in protein folding, red the proteins involved in chloroplast proteolytic degradation, orange the components of the chloroplast import machinery, blue the proteins involved in the ubiquitin–proteasome system (UPS) and green the Q28 or Q69 proteins. **c**, Scheme indicating the subcellular localization of selected common interactors of Q69 and Q28. **d**, Co-IP with GFP and control IgG antibodies in Q69 seedlings followed by western blotting against the chloroplast protease subunits ClpP4 and FtsH2/8. The results represent three independent experiments. **e**, Q28 and Q69 distribution in mesophyll cells of 7-day-old cotyledons. Images show citrine fluorescence (green) and chloroplast autofluorescence (red). Scale bar, 5 μm. The results represent four independent experiments. Source data

**Extended Data Fig. 4:**
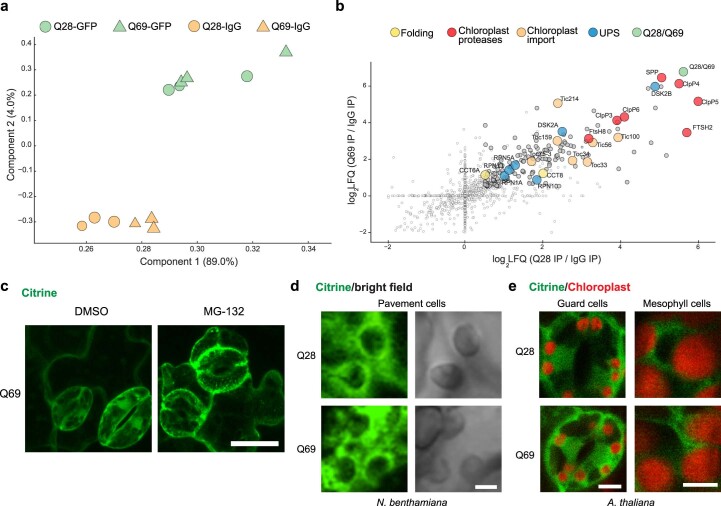
Ectopically expressed Q28 and Q69 share common protein interactors in plants. **a**, Principal component analysis (PCA) groups Q28 and Q69 interactors (*n* = 3 biological replicates). **b**, Scatterplot of protein enrichments in Q28/Q69 co-immunoprecipitation (co-IP) from Q28 and Q69 7-day-old seedlings. Gray and colored circles indicate significance after correction for multiple testing (*n* = 3 biological replicates, False Discovery Rate (FDR) < 0.05 was considered significant). Yellow circles: proteins involved in protein folding, red: proteins involved in chloroplast proteolytic degradation, orange: components of the chloroplast import machinery, blue: proteins involved in the ubiquitin-proteasome system (UPS), green: Q28 or Q69 proteins. **c**, Q69 aggregates upon proteasome inhibition. 7-day-old Q69 seedlings were transferred to liquid MS supplemented with DMSO or 50 μM MG-132. Images were taken after 8 hours of treatment. Scale bar: 20 μm. **d**, Citrine-Q28 and Citrine-Q69 distribution in *N. benthamiana* pavement cells from leaves analyzed at 6 days post-agroinfiltration. Bright field images of chloroplasts are shown. Scale bar: 5 μm. **e**, Citrine-Q28 and Citrine-Q69 distribution in guard and mesophyll cells of 7-day-old transgenic Arabidopsis seedlings. Images show Citrine fluorescence (green) and chloroplast autofluorescence (red). Scales bar: 5 μm. In c-e, the images are representative from three independent experiments.

To investigate the mechanisms underlying the enhanced ability of plants to prevent polyQ aggregation under normal conditions, we performed pulldown experiments of Q28 and Q69 in *Arabidopsis* sp. followed by label-free proteomics. Q28 and Q69 were the most enriched proteins in the corresponding transgenic plants after immunoprecipitation, thereby validating our assay (Fig. 2a,b and Supplementary Table 2). Hierarchical clustering analysis revealed a similar network of interactions between Q69 and Q28 lines (Extended Data Fig. 4a,b). Thus, the plant proteostasis interactors did not differ between the relatively long Q28 and Q69 stretches, considering that Q24 represents the longest polyQ stretch in *Arabidopsis* proteins.

Among the proteins interacting with Q28 and Q69, we found several factors involved in cytosolic protein folding and the ubiquitin–proteasome system (Fig. 2a–c and Supplementary Table 2). For instance, we identified subunits of the T-complex protein ring complex (TRiC)/chaperone containing TCP-1 (CCT) complex (Fig. 2a–c), a chaperonin that reduces the accumulation of polyQ aggregates in human cells and *C. elegans* models^5,10^. Moreover, we detected the ubiquitin-binding receptors DSK2A and DSK2B as interactors of Q28 and Q69 in *Arabidopsis* sp. (Fig. 2a–c). Importantly, DSK2 (also known as ubiquilin) suppresses polyQ-expanded protein aggregation and toxicity in animal models of Huntington’s disease^28^. Consistent with other interactome studies in mammalian cells^29^, we identified several proteasome subunits as polyQ interactors in plants (Fig. 2a–c). In animal cells, proteasome inhibition leads to the aggregation of polyQ-expanded proteins^9^. Similarly, we observed Q69 aggregation when plants were exposed to the proteasome inhibitor MG-132 (Extended Data Fig. 4c).

In addition to cytosolic proteostasis components, our interactome analysis revealed that polyQ proteins bind to chloroplast-specific proteins such as the SPP. We also found several components of TOC/TIC, the chloroplast import machinery, as well as the protease complexes Clp and FtsH (Fig. 2a–d). Moreover, confocal microscopy analyses indicated that both Q28 and Q69 localize around the chloroplasts (Fig. 2e and Extended Data Fig. 4d,e). These findings suggest a potential link between chloroplasts and polyQ proteostasis in plants.

### Chloroplast disruption causes cytosolic polyQ aggregation

Most chloroplast proteins are encoded by the nuclear genome and synthesized in the cytosol as unfolded protein precursors (or pre-proteins), which are imported into chloroplasts by the TOC/TIC machinery. Pre-proteins contain an unstructured/unfolded amino-terminal transit peptide^30,31^ that is recognized by the TOC/TIC complex and transported into the stroma for proteolytic processing by proteases^32,33^. The protease complexes Clp and FtsH also degrade damaged and misfolded proteins, thus maintaining chloroplast proteostasis^32,33^. Notably, the interactome of both Q28 and Q69 was enriched for subunits of the TOC/TIC import machinery, as well as the Clp and FtsH proteases (Fig. 2a–c).

**Extended Data Fig. 5:**
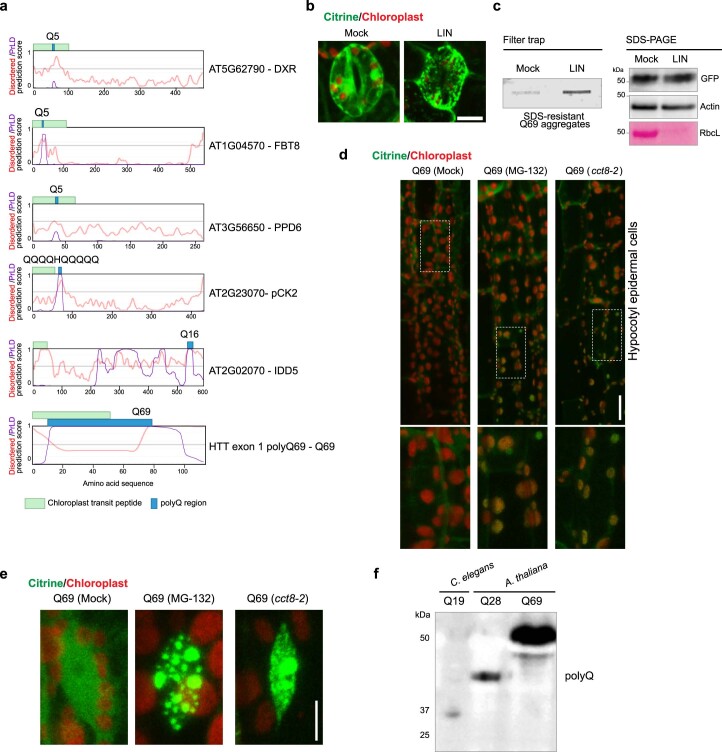
Q69 aggregates upon long-term LIN treatment. **a**, Prion-like domain (PrLD) amino score (purple line) was predicted using PLAAC (http://plaac.wi.mit.edu/). Protein unstructured score (red line) was predicted with IUPred3 (https://iupred.elte.hu). Green boxes represent the predicted transit peptide according to ChloroP 1.1. Blue boxes indicate the longest polyQ domain found in the amino acid sequence. Annotated chloroplast proteins were selected from Supplementary Data 1. **b**, Representative confocal microscopy images showing Q69 distribution in 7-day-old seedlings grown and germinated in media supplemented with LIN 15 μM or mock solution. Images taken in stomata cells. Scale bar: 10 μm. Representative of three independent experiments. **c**, Filter trap and SDS-PAGE analysis of seedlings from panel b. Representative blots of 3 independent experiments. **d**, Q69 can be targeted to chloroplasts upon impairment of cytosolic proteostasis. Confocal analysis of 7-day-old Q69, Q69-MG132-treated, and Q69 in the mutant background *cct8-2*. Q69-MG-132-treated plants were germinated and grown in MS media supplemented with 50 μM MG-132. Cells from hypocotyls are shown. Dotted rectangles indicate the zoomed panels shown below. Scale bar: 20 μm. Representative of three independent experiments. **e**, Q69 distribution in the nucleus from samples shown in panel d. Scale bar: 5 μm. Representative of three independent experiments. **f**, Immunoblot analysis of *Caenorhabditis elegans* expressing Q19, and constitutive transgenic Arabidopsis plants expressing Q28 and Q69. Immunoblot shows that anti-polyQ antibody can recognize different polyQ repeat sizes. Representative of two independent experiments. Source data

We hypothesized that polyQ proteins can be recognized by the chloroplast import machinery. First, we analyzed the endogenous *Arabidopsis* proteome, searching for polyQ stretches in annotated chloroplast proteins (Supplementary Table 1). From the nucleus-encoded chloroplast list of proteins with polyQ stretches, we found that five out of these proteins have the polyQ repeats close to the N-terminal chloroplast transit peptide (Extended Data Fig. 5a). Prediction software indicated that the polyQ stretches from chloroplast proteins are embedded in prion-like domains or intrinsically disordered regions (Extended Data Fig. 5a). Likewise, the Q69 protein, which has a large prion-like/disorder domain, was also predicted to be a chloroplast protein (Extended Data Fig. 5a).

**Fig. 3:**
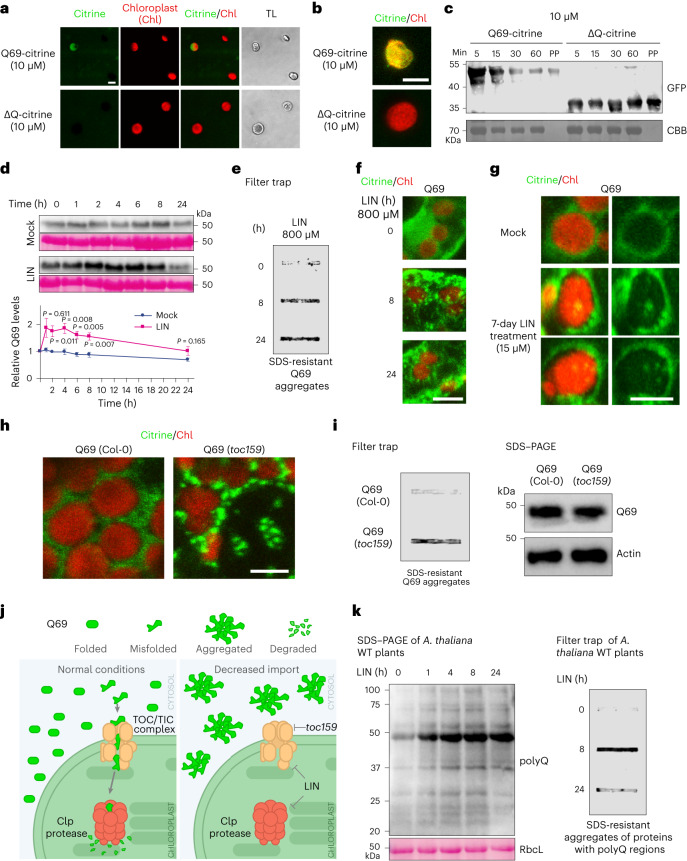
Reduced chloroplast proteostasis leads to Q69 aggregation. **a**, Confocal microscopy of isolated chloroplasts incubated with 10 μM recombinant poly(Q69)-HTTexon1 fused to citrine (Q69-citrine) or control HTTexon1-citrine lacking the polyQ stretch (∆Q-citrine) at 25 °C under light for 30 min. TL, transmitted light. **b**, Higher magnification of isolated chloroplasts. Scale bar, 5 μm in **a** and **b**. Images represent four independent experiments. **c**, Western blotting with anti-GFP antibody of isolated chloroplasts incubated with 10 μM Q69-citrine or ∆Q-citrine for the indicated times. Coomassie Brilliant Blue (CBB) is the loading control; 0.1 μM of purified protein (PP) Q69-citrine or ∆Q-citrine was loaded for reference. The results represent four independent experiments. **d**, Western blotting with anti-GFP of 7-day-old Q69 plants treated with mock or 800 μM LIN. RbcL is the loading control. The results represent four independent experiments. The graph shows relative Q69 protein levels to time point 0 (mean ± s.e.m. of four independent experiments, except mock 24 h = 3 experiments). Statistical comparisons were made using two-tailed Student’s *t*-test for unpaired samples. **e**, Filter trap with anti-GFP of Q69 aggregation on LIN treatment for the indicated times. The results represent three independent experiments. **f**, Representative images of Q69 aggregation in stomata from cotyledons after LIN treatment. Images show citrine (green) and chloroplast autofluorescence (red). **g**, Citrine fluorescence (green) within the chloroplast (red) of epidermal hypocotyl cells. **h**, Representative images of mesophyll cells of 7-day-old seedlings showing Q69 distribution on Col-0 and *toc159* backgrounds. Scale bar, 5 μm in **f**–**h**. Images represent three independent experiments. **i**, Filter trap and SDS–PAGE analysis of the samples presented in **h**. Actin is the loading control. The results represent two independent experiments. **j**, Schematic model of chloroplast-mediated regulation of poly(Q69). Under normal conditions, Q69 distributes homogeneously around the chloroplasts, whereas misfolded/unstructured variants are imported to the chloroplast for degradation. When chloroplast import and degradation are impaired, misfolded Q69 accumulates in the cytosol forming aggregates. **k**, Western blotting and filter trap analysis with anti-polyQ antibody of 7-day-old WT plants treated with 800 μM LIN for the indicated hours. RbcL is the loading control. The results represent three independent experiments. Source data

To assess whether polyQ-expanded proteins are imported and degraded within the chloroplast, we incubated isolated chloroplasts with purified recombinant poly(Q69)-HTTexon1 fused to the fluorescent tag citrine (Q69-citrine). We found that isolated chloroplasts import Q69-citrine, but not control HTTexon1-citrine lacking the polyQ stretch (∆Q-citrine) (Fig. 3a,b). In addition, isolated chloroplasts degraded Q69-citrine over time, whereas the levels of ∆Q-citrine remained stable (Fig. 3c). To investigate the impact of chloroplasts on polyQ proteostasis in vivo, we treated plants with lincomycin (LIN), which impairs both chloroplast protein import and Clp protease-mediated degradation^34,35^. When we transferred 7-day-old Q69 seedlings to liquid medium supplemented with 800 μM LIN, we observed a rapid accumulation and aggregation of Q69 (Fig. 3d–f). After 24 h of treatment with 800 μM LIN, Q69 remained aggregated but its soluble levels were reduced (Fig. 3d–f). These results suggest that blocking chloroplast import and Clp-mediated degradation initially increases Q69 levels, leading to its aggregation. Eventually, the prolonged aggregation induced by acute LIN treatment reduces the levels of monomeric, soluble Q69 (Fig. 3d–f). Notably, treating plants with lower concentrations of LIN (15 μM) for extended periods of time (7 d) also triggered Q69 aggregation (Extended Data Fig. 5b,c). During long-term LIN treatment, we detected citrine fluorescence within some chloroplasts (Fig. 3g), providing further evidence that Q69 can be imported into these organelles. Similarly, we also observed Q69 aggregates in *toc159* plants, a mutant line with altered chloroplast import (Fig. 3h,i).

In human cells and animal models, the cytosolic TRiC/CCT chaperonin and the ubiquitin–proteasome system prevent polyQ-expanded aggregation^5,9,10,29^. Importantly, genetic impairment of cytosolic folding through loss of the TRiC/CCT complex and prolonged proteasomal inhibition allowed us to detect Q69-citrine fluorescence in chloroplasts as well as the formation of nuclear condensates/aggregates (Extended Data Fig. 5d,e). Collectively, our data suggest that Q69 can be targeted to different subcellular compartments and chloroplasts may play a major role in preventing the accumulation of Q69 aggregates in the cytosol (Fig. 3j). Supporting this hypothesis, when chloroplasts were transiently impaired on LIN treatment, cytosolic Q69 levels rapidly increased, surpassing a threshold that triggers the formation of cytosolic aggregates (Fig. 3d–f).

Intrigued by the interplay between chloroplast proteostasis and the regulation of Q69 aggregation, we asked whether LIN treatment also promotes the aggregation of endogenous polyQ proteins in *Arabidopsis* sp. (Supplementary Table 1). To this end, we used a polyQ antibody that specifically recognizes proteins containing polyQ stretches (Extended Data Fig. 5f). Remarkably, treatment of wild-type (WT) *Arabidopsis* plants with LIN caused a strong accumulation and aggregation of endogenous polyQ proteins, indicating a central role of chloroplasts in polyQ proteostasis (Fig. 3k).

### SPP reduces polyQ aggregation in human cells and *C. elegans*

**Fig. 4:**
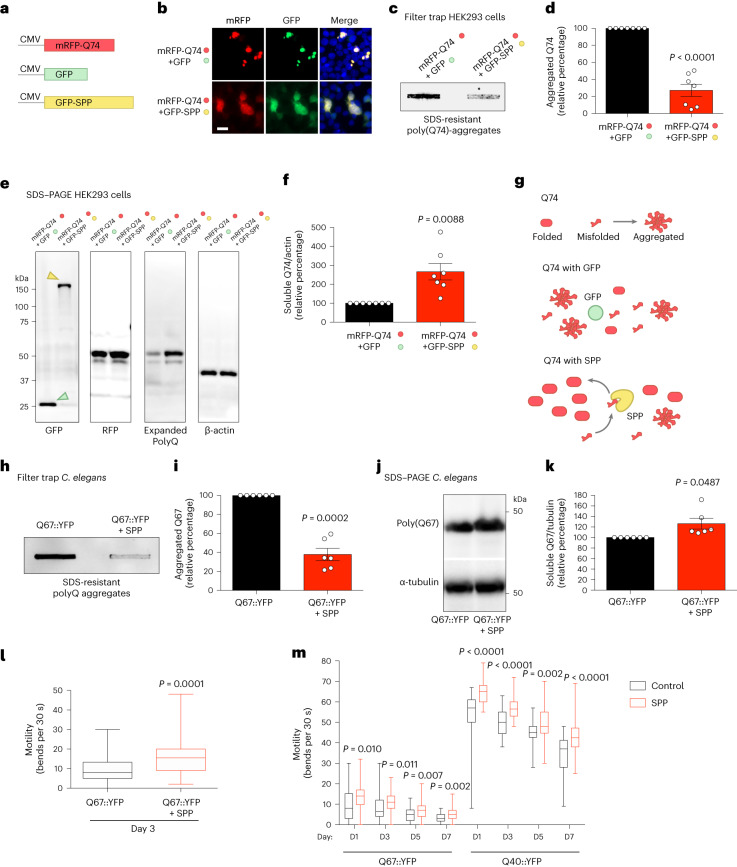
Synthetic SPP reduces polyQ-expanded aggregation in human cells and *C. elegans*. **a**, Constructs for protein expression in human HEK293 cells. **b**, Images of HEK293 cells co-transfected with mRFP-Q74 and either control GFP or GFP–SPP. Blue signal corresponds to cell nuclei (Hoechst 33342). Scale bar, 20 μm. The results represent three independent experiments. **c**, Filter trap with anti-mCherry antibody of SDS-insoluble, aggregated mRFP-Q74 in HEK293 cells. The results represent seven independent experiments. **d**, Relative percentage values of aggregated mRFP-Q74 to samples expressing mRFP-Q74 + control GFP (mean ± s.e.m.; *n* = 7 independent experiments). **e**, Western blotting of HEK293 cells with anti-GFP antibody to detect control GFP (27 kDa, green arrowhead) and SPP–GFP (~163 kDa, yellow arrowhead). Anti-mCherry and anti-polyQ-expanded antibodies were used to detect soluble mRFP-Q74 levels. *β*-Actin is the loading control. The results represent seven independent experiments. **f**, Relative percentage values of soluble mRFP-Q74 (corrected for *β*-actin loading control) to samples expressing mRFP-Q74 + control GFP (mean ± s.e.m.; *n* = 7 independent experiments). **g**, Schematic model of synthetic SPP effects in preventing mRFP-Q74 aggregation. **h**, Filter trap with anti-expanded polyQ antibody of day 3 adult worms expressing neuronal polyQ67::YFP (yellow fluorescent protein). The results represent six independent experiments. **i**, Relative percentage values of aggregated poly(Q67) levels in *C. elegans* on SPP expression to control Q67 (mean ± s.e.m.; *n* = 6 independent experiments). **j**, Western blotting of soluble polyQ67::YFP levels (detected by anti-GFP antibody, where YFP is yellow fluorescent protein) in day 3 adult worms. α-tubulin is the loading control. The results represent six independent experiments. **k**, Relative percentage of soluble poly(Q67) levels in worms on SPP expression (corrected for *α*-tubulin levels) to control Q67 (mean ± s.e.m.; n = 6 independent experiments). **l**, Thrashing movements in day 3 adult poly(Q67)-expressing worms over a 30-s period (*n* = 50 worms per condition from three independent experiments). **m**, Thrashing movements in poly(Q67)- and poly(Q40)-expressing worms over a 30-s period at the indicated days (D) of adulthood (*n* = 50 worms per condition from two independent experiments). In **l**–**m**, the box plots represent the 25th–75th percentiles, the line depicts the median and the whiskers show the minimum–maximum values. Statistical comparisons were made using two-tailed Student’s *t*-test for paired (**d**, **f**, **i** and **k**) or unpaired samples (**l** and **m**). Source data

Besides Q69 itself, the SPP stood out as the most enriched protein after immunoprecipitation of poly(Q69) in plants (Fig. 2b). Similarly, SPP was also one of the most enriched interactors of Q28 (Fig. 2a). SPP binds to pre-proteins and cleaves their chloroplast transit peptide through a single endoproteolytic step^36^. In addition, SPP is upregulated and binds to unstructured peptides to counteract the loss of chaperone capacity in plants, suggesting a role for SPP in preventing folding stress^37^. To explore whether SPP can function in human cells to decrease polyQ-expanded aggregation, we co-transfected human HEK293 cells with monomeric RFP-HTTexon1-Q74 (mRFP-Q74) and a synthetic SPP (without chloroplast transit peptide and human codon optimized) fused to green fluorescent protein (GFP) in the N terminus (GFP–SPP) (Fig. 4a,b). Microscopy analysis revealed that expression of GFP–SPP reduces aggregation of mRFP-Q74 when compared with cells co-expressing mRFP-Q74 and control GFP (Fig. 4b). By filter trap assay, we confirmed that ectopic expression of GFP–SPP reduces the amounts of sodium dodecylsulfate (SDS)-insoluble mRFP-Q74 (Fig. 4c,d).

**Extended Data Fig. 6:**
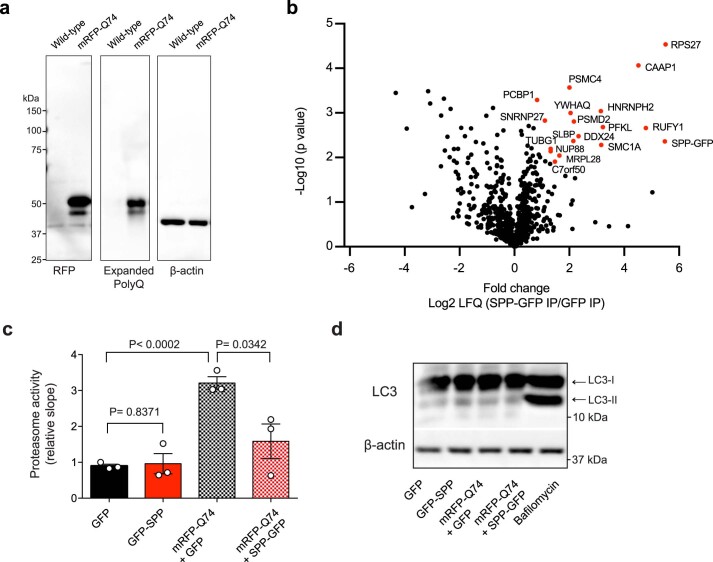
Synthetic SPP-GFP interacts with endogenous proteins of HEK293 cells. **a**, Western blot of wild-type HEK293 cells and HEK293 cells transfected with mRFP-Q74. Anti-mCherry and anti-polyQ-expanded antibodies were used to detect soluble mRFP-Q74. Both anti-mCherry and anti-polyQ-expanded antibodies detected two common bands of soluble mRFP-Q74 with different electrophoretic mobilities, that is the most intense band of ~55 kDa and another band of ~43kD. *β*-actin is the loading control. Representative of 9 independent experiments. **b**, Volcano plot of the interactome of synthetic SPP-GFP in HEK293 cells. Graph represents the –log_10_ (P-value) of a two-tailed *t*-test plotted against the log_2_ fold change of protein label-free quantification (LFQ) values from co-immunoprecipitation experiments using anti-GFP antibody. HEK293 cells expressing SPP-GFP were compared with HEK293 cells expressing control GFP. Red dots indicate significant interactors of SPP-GFP when compared to control GFP after correction for multiple testing. Student’s *t*-test (n = 3 biological replicates), False Discovery Rate (FDR)-adjusted *P* value (q value) <0.05 was considered significant. **c**, Chymotrypsin-like proteasome activity in HEK293 cells (relative slope to control GFP HEK293 cells). Graph represents the mean ± s.e.m. of three independent experiments. All the statistical comparisons were made by two-tailed Student’s *t*-test for unpaired samples. **d**, Western blot of HEK293 cells with anti-LC3 antibody to monitor autophagy flux. LC3-I is conjugated to phosphatidylethanolamine to form LC3-II, which amounts reflect the number of autophagosomes and autophagy-related structures. As a control, we treated wild-type HEK293 cells with 250 nM bafilomycin A (8 h), an inhibitor of autophagy that greatly increases the amount of LC3-II. β-actin is the loading control. Representative of three independent experiments. Source data

Considering the robust decline in mRFP-Q74 aggregation induced by ectopic expression of SPP, we investigated whether SPP concomitantly increases the levels of soluble mRFP-Q74. Given that insoluble/aggregated polyQ-expanded proteins do not enter the running gel, a western blotting assay provides a tool to quantify the levels of soluble, monomeric polyQ proteins^38,39^. The mRFP-Q74 protein can be detected by western blotting using antibodies that recognize either the mRFP tag (anti-mCherry antibody) or the expanded polyQ stretch (anti-polyQ-expansion diseases marker)^9,40,41^. Western blotting analysis revealed two common bands of soluble mRFP-Q74 with different electrophoretic mobilities detected by both antibodies, that is, a more intense band of ~55 kDa and another band of ~43 kDa (Extended Data Fig. 6a and Fig. 4e). Notably, the levels of soluble mRFP-Q74 increased on ectopic expression of SPP (Fig. 4e,f), which correlates with the decreased amounts of aggregated mRFP-Q74 observed by filter trap assay (Fig. 4c,d). Although we cannot entirely rule out the possibility of SPP also cleaving mRFP-Q74 in human cells, our data primarily support SPP preventing the self-assembly of mRFP-Q74 into aggregates, resulting in elevated levels of the monomeric fraction (Fig. 4g).

To investigate whether SPP could affect other pathways and possibly diminish its therapeutic potential, we performed an interactome assay comparing GFP–SPP with control GFP in WT HEK293 cells (Supplementary Table 3). We found that GFP–SPP interacts with 17 proteins of the endogenous HEK293 proteome, including 9 RNA-binding proteins involved in different processes, such as splicing and translation (DDX24, HNRNPH2, RPS27, MRPL28, PCBP1, C7orf50, SLBP, SMC1A and SNRNP27) (Extended Data Fig. 6b and Supplementary Table 3). Therefore, it is important to consider the possibility of an off-target effect of synthetic SPP on RNA metabolism. In addition to RNA-binding proteins, we found that SPP interacts with proteasome subunits (PSMD2 and PSMC4) in human cells (Extended Data Fig. 6b and Supplementary Table 3). Given that polyQ-expanded proteins also interact with proteasome subunits and can be degraded by the proteasome^9,29^ (Fig. 2a–c), we asked whether ectopic expression of SPP influences proteasome activity. However, we observed that SPP does not increase proteasome activity in control HEK293 cells (Extended Data Fig. 6c). On the other hand, the expression of mRFP-Q74 triggered proteasome activity (Extended Data Fig. 6c), which suggests a compensatory mechanism to cope with proteotoxic stress resulting from the accumulation of polyQ aggregates^42^. However, expression of SPP partially decreased the induction of proteasome activity in these cells (Extended Data Fig. 6c), probably because SPP reduces poly(Q74) aggregation and subsequent proteostasis collapse (Fig. 4b–d). Although this decline in proteasome activity could contribute to the elevated levels of mRFP-Q74 detected by western blotting (Fig. 4e,f), it cannot explain the suppression of mRFP-Q74 aggregation induced by SPP (Fig. 4b–d). The autophagy–lysosome pathway can also terminate protein aggregates, but we did not observe changes in the autophagic flux on SPP expression (Extended Data Fig. 6d). Taken together, our results indicate that synthetic SPP does not activate the two major proteolytic systems.

Besides proteolytic systems, we assessed whether SPP induces conformational changes across the proteome by limited proteolysis–mass spectrometry (LiP–MS)^43^. In the LiP–MS method, protein extracts are first subjected to protease digestion with the nonspecific proteinase K for a short time under native conditions, followed by complete digestion with the sequence-specific trypsin under denaturing conditions. This sequential protease treatment generates conformation-specific peptides, depending on the structural features of the protein, for MS analysis^43^. However, due to the inability of proteinase K to cleave after glutamine residues, the expanded polyQ stretch remains resistant to this protease, regardless of its conformational state^44^. Although LiP–MS cannot be used to distinguish changes in Q74 structure, we were able to assess thousands of other proteins (Supplementary Table 4). However, we did not find significant off-target effects of protein structure on SPP expression after correction for multiple testing (Supplementary Table 4).

To assess the potential ameliorative effects of SPP in vivo, we used *C. elegans* models expressing polyQ-expanded repeats in neurons^27^. In these animals, polyQ-expanded peptides form aggregates throughout the nervous system, with a pathogenic threshold of 40 repeats^27^. Similar to human HEK293 cells, we found that ectopic expression of SPP reduces the amounts of neuronal Q67 aggregates while slightly increasing the levels of monomeric Q67 (Fig. 4h–k). The accumulation of polyQ aggregates leads to neurotoxicity and subsequent decline in the motility of the worms, resembling a disease-like phenotype^9,10,27,41,45,46^. The severity and onset of neuronal deficits correlate with the length of the polyQ repeats^27^. As such, poly(Q67)-expressing worms exhibit severe loss of motility even at an early age^27^. Notably, ectopic expression of SPP improved the impaired motility phenotype of young Q67-expressing worms (Fig. 4l). Thus, our data indicate that SPP can prevent polyQ aggregation and subsequent neurotoxicity in *C. elegans*. To evaluate the effects of SPP in the context of aging, we examined *C. elegans* expressing poly(Q40) repeats, a less aggressive polyQ stretch^27^. We observed that ectopic expression of SPP attenuates the decline in motility of poly(Q40)-expressing worms during aging (Fig. 4m).

**Extended Data Fig. 7:**
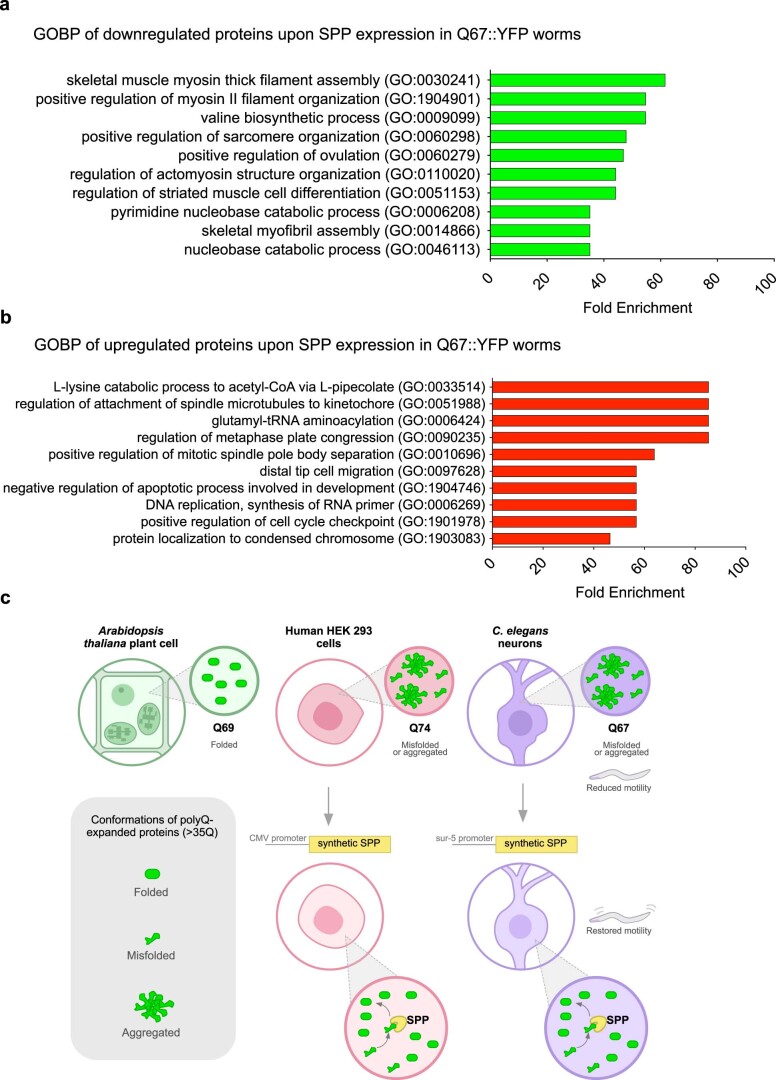
Besides reducing polyQ-expanded aggregation, SPP expression leads to changes in the total levels of different proteins in Q67-expressing worms. **a-b**, Gene Ontology Biological Process (GOBP) analysis of downregulated (a) and upregulated (b) proteins in 3-day adult Q67::YFP worms + SPP compared with control Q67::YFP worms (FDR < 0.05, Analysis tool: PANTHER Gene Ontology Resource, release 2023-06-11). Ten of the most enriched GOBP terms are shown. Please see Supplementary Data 5 for the complete list of enriched GOBP terms. **c**, To explore how plants prevent toxic protein aggregation, we expressed an aggregation-prone human huntingtin (Q69) fragment in *Arabidopsis thaliana*. In contrast to *C. elegans* and mammalian transgenic models, we found that Arabidopsis plants suppress toxic Q69 aggregation and do not display adverse or harmful effects. Proteomic experiments identified the chloroplast stromal processing peptidase (SPP) as one of the strongest interactors of Q69 in plant cells. Using synthetic biology and ectopically expressing SPP in human HEK293 cells and *C. elegans* disease models, we reduced the toxic aggregation of polyQ-extended proteins. Thus, our findings indicate a promising plant synthetic biology approach towards developing therapies for human neurodegenerative diseases that involve protein aggregation, particularly Huntington’s disease. However, further studies are required to define potential off-target effects of synthetic SPP expression in human cells.

To investigate potential off-target effects of SPP expression in *C. elegans*, we performed quantitative proteomics analysis of poly(Q67)-expressing worms (Supplementary Table 5). Although we were unable to quantify poly(Q67) by proteomics due to lack of identifiable peptides after tryptic digestion in its sequence, we could quantify nearly 1,400 other proteins. We found that SPP expression leads to a decrease in the levels of 163 proteins in Q67-expressing worms, whereas 168 proteins were upregulated (Supplementary Table 5). The downregulated proteins were enriched for factors involved in muscle myosin filament assembly, valine biosynthesis and nucleobase catabolism (Extended Data Fig. 7a and Supplementary Table 5). On the other hand, the upregulated proteins were enriched for factors involved in l-lysine catabolism, glutamyl-transfer RNA aminoacylation, mitotic spindle regulation, DNA replication and cell cycle (Extended Data Fig. 7b and Supplementary Table 5). Although some of these changes might be a consequence of the beneficial effects of SPP in preventing polyQ aggregation and neurodegeneration, we cannot rule out the possibility of off-target effects. Together, our results across different species suggest that synthetic SPP holds promise as a potential therapeutic approach for the treatment of Huntington’s disease and other polyQ disorders, but potential off-target effects should be considered.

## Discussion

To our knowledge, unlike mammals, plants do not experience proteinopathies caused by the abnormal aggregation of polyQ proteins. The presence of chloroplasts in plant cells potentially expands the repertoire of proteostasis components, such as chaperones and proteases, which may counteract cytosolic toxic protein aggregation. In non-plant models, the proteostasis network of subcellular compartments like the endoplasmic reticulum and nucleus can clear misfolded proteins that would otherwise be prone to aggregation when accumulated in the cytosol^41,47,48^. Moreover, aggregated cytosolic proteins are disentangled on the mitochondrial surface and subsequently imported for degradation by mitochondrial proteases^49–51^. Considering the numerous similarities between mitochondria and chloroplasts, it is plausible that parallel mechanistic pathways exist, awaiting discovery through a better understanding of chloroplast biology. Along these lines, we find that chloroplasts import and degrade cytosolic poly(Q69)-expanded protein through Clp and FtsH proteases. Conversely, impairing chloroplast import triggers the formation of Q69 aggregates in the cytosol. The unstructured configuration of Q69 protein led us to speculate that the polyQ region could be recognized as an unfolded N-terminal transit peptide in a pre-protein. Indeed, in vitro import assays demonstrate that Q69 protein is imported into chloroplasts, whereas removal of the polyQ stretch hinders the import process.

We identified SPP, a protein that binds and cleaves chloroplast transit peptides, as the most enriched interactor of Q69. It has been proposed that SPP does not recognize a strict sequence motif for cleaving transit peptides, but rather recognizes transition between unfolded and folded regions of chloroplast pre-proteins^36,37,52^. Together, our data suggest that aggregation-prone Q69 could be recognized by the chloroplast import machinery for further processing by SPP. Similarly, the human signal peptidase complex, which removes endoplasmic reticulum signal peptides, supports the degradation of misfolded proteins^53^.

The accumulation of misfolded/aggregated proteins, leading to cell dysfunction and death, is a hallmark of age-related neurodegenerative diseases^54,55^. Given the interaction of Q69 with SPP and the absence of aggregation in plants with functional chloroplasts, we hypothesized that plant-derived SPP could be a potential treatment for human polyQ-related neurodegenerative diseases. In recent years, there has been increasing interest in using plant proteins as therapeutic agents for human diseases. For instance, nanothylakoids containing photosynthetic proteins have been introduced into animal cells to restore anabolism in certain diseases and supply cells with ATP and NADPH^56^. Moreover, ectopic expression of plant RDR1 can inhibit cancer cell proliferation^57^. In our study, we discovered that synthetic SPP can be expressed in human cells and worm models to prevent polyQ aggregation (Extended Data Fig. 7c). Further work is required to elucidate the detailed molecular mechanisms by which SPP prevents the aggregation of polyQ-expanded proteins, because SPP does not appear to promote degradation in human and worm models. Moreover, it will be fascinating to explore whether synthetic SPP, or other plant proteins, can also prevent aggregation of distinct disease-related proteins, such as mutant TDP-43 or FUS variants, which cause amyotrophic lateral sclerosis^1^. Beyond SPP, our interactome data of polyQ-expanded proteins in plants provide a plethora of potential therapeutic targets that can be explored in future studies.

Although our findings raise the intriguing prospect of utilizing SPP and other chloroplast proteins as therapeutic agents, it is important to consider the possible off-target effects. We observed that synthetic SPP interacts with several RNA-binding proteins in human cells. In addition, expression of SPP changes the total levels of >300 proteins in *C. elegans*. Therefore, although SPP may alleviate disease-related protein aggregation and neurodegeneration, it might also have unintended consequences that require further investigation.

## Methods

### Plant material and constructs

*A. thaliana* lines of Columbia-0 (Col-0) ecotype were employed, including WT, *toc159* (refs. ^58,59^) and *cct8-2* (ref. ^60^). Seeds underwent surface sterilization and germination on solid 0.5× Murashige and Skoog (M&S) medium with vitamins, lacking sucrose. Plants were incubated in a growth chamber at 22 °C under long-day conditions (or otherwise indicated) and supplemented with 17-β-estradiol (Sigma-Aldrich) when specified. The MG-132 (Bio-Techne) and LIN (Sigma-Aldrich) treatments were performed on liquid 0.5× M&S medium. We used FIJI (ImageJ) to measure root length in 7-day-old seedlings grown on vertical agar plates.

For cloning, we used Gateway BP and LR Clonase II Enzyme mix (Thermo Fisher Scientific). *Q28* and *Q69* genes were generated using the plasmid pEGFP-Q74 (ref. ^61^), with different polyQ lengths amplified and sequenced. These genes were subcloned into the entry vector pDONR221, then into vector pMpGWB105 (glutamine (Q) constructs). Supplementary Table 6 contains details about the primers used in the present study. Citrine-Q28 and citrine-Q69 were amplified from pMpGWB105:Q28/Q69 plasmids and subcloned into entry vector pDONR221, then into destination vector pMDC7 (iQ constructs). *Arabidopsis* transgenic plants were generated through the floral dip method^62^. The *35S:Citrine-Q69* transgene was introduced into the *toc159* or *cct8-2* mutant background by crossfertilization.

For flowering time experiments, plants were grown in short-day conditions and rosette leaf numbers were counted until a visible bolt formed. Photosynthetic activity was assessed using the M-Series PAM fluorometer, with analysis conducted via ImagingWin (v.2.41a) software (Heinz Walz GmbH). For heat shock assays, a single plate containing 7-day-old WT, Q28 and Q69 was covered with aluminum foil at 45 °C (or 37 °C) for specified durations. The mock plate remained under control conditions covered with aluminum foil. Heat-treated plates were returned to 22 °C under light conditions. Microscopy images were captured using a Meta 710 Confocal Microscope with laser ablation 266 nm (Zeiss) using the same parameters between experiments.

### Gene expression analysis

Total RNA was extracted from plant tissues using the RNeasy Plant Mini Kit (QIAGEN). Subsequently, complementary DNA was synthesized using the qScript Flex cDNA synthesis kit (Quantabio). SYBR green real-time quantitative (q)PCR experiments were performed with a 1:20 dilution of cDNA using a CFC384 Real-Time System (BioRad). Data were analyzed with the comparative 2ΔΔ*C*_t_ method using the geometric mean of *Ef1α* and *PP2A* as housekeeping genes. Supplementary Table 6 contains details about the primers used for qPCR.

### Analysis of the *Arabidopsis* polyQ proteome

The *Arabidopsis* proteome was obtained from UniProt and filtered to find proteins with five or more consecutive glutamine repeats and annotated chloroplast proteins. Prion-like domains were identified in selected protein sequences using PLAAC software (http://plaac.wi.mit.edu)^63^. A minimum length for prion-like domains (L core) was set at 60 and parameter α was set at 50. To identify intrinsically disordered regions, we used IUPred3 software (https://iupred.elte.hu)^64^.

### Protein expression and purification

Chemically competent *Escherichia coli* BL21(DE3) cells were transformed with pGEX-6P-1 vector (GE Healthcare), carrying mtHTT-Exon1-polyQ69-citrine (Q69-citrine) and HTT-Exon1-citrine (ΔQ-citrine) constructs. Cultures were grown at 37 °C before protein expression was induced with 0.25 mM isopropyl 1-thio-β-d-galactopyranoside at 18 °C for 20 h. After harvesting and ultrasound sonication, lysates were centrifuged (25,000*g*, 4 °C, 1 h). Recombinant proteins were purified by glutathione *S*-transferase (GST) affinity chromatography using a Qtathione-Sepharose 4B column (Cytiva). Proteins were eluted with 20 mM reduced glutathione and 5 mM dithiothreitol (DTT) in phosphate-buffered saline (PBS), pH 8. Then, free glutathione was removed from the protein solution by dialysis and the GST-fusion tag was removed with HRV 3C Protease, followed by another GST affinity chromatography. We assessed protein purity by SDS–polyacrylamide gel electrophoresis (PAGE) and concentrated pure fractions by spin filtration for import assays.

### Chloroplast isolation and protein import

Incubation occurred at 25 °C under light, halted at 5, 15, 30 and 60 min. Samples were stopped with ice-cold EDTA-containing buffer, centrifuged and chloroplast pellets were resuspended in 2× Leammli buffer. SDS–PAGE and western blotting with anti-GFP antibody-assessed time points. Microscopy used the 30-min import reaction on a microscope slide. Chloroplasts were isolated from 12-day-old *Arabidopsis* seedlings as described^65^. For each 600 µl of import reaction, we used 10 million chloroplasts supplemented with 120 µl of 10× HMS buffer (500 mM Hepes, 30 mM MgSO_4_ and 3.0 M sorbitol, pH 8.0), 12 µl of 1 M gluconic acid (potassium salt), 6 µl of 1 M NaHCO_3_, 6 µl of 20% (w/v) bovine serum albumin, 30 µl of 100 mM MgATP and 10 µM of Q69-citrine or ΔQ-citrine. To stop the reaction at different time points, we transferred 130 µl to a fresh tube with ice-cold import stop buffer (50 mM EDTA dissolved in 1× HMS buffer) and all the tubes were retained on ice until the time course was completed. All samples were centrifuged (12,000*g*, 30 s) and pellets containing the chloroplasts were resuspended in 25 µl of 2× Leammli buffer for western blotting analysis. For microscopy imaging, we pipetted 60 µl of the 30-min import reaction on to a microscope slide.

### HEK293 cell transfection

CMV:pEGFP-Q74 plasmid was digested (BglII, BamHI) to remove *Q74* gene and generate *pEGFP* (*CMV:GFP*). Synthetic *SPP* (AGI locus code AT5G42390) gene, codon optimized, lacking chloroplast transit peptide, was made by Twist Bioscience. *CMV:GFP-SPP* was generated by cloning the synthetic *SPP* gene into pDEST-CMV-N-GFP vector using Gateway technology.

HEK293 cells (American Type Culture Collection (ATCC), HEK293T/17, catalog no. CRL-11268) were cultured on gelatin-coated plates in Dulbecco’s modified Eagle’s medium (DMEM) supplemented with 10% fetal bovine serum and 1% MEM Non-essential Amino Acid solution (Gibco) at 37 °C. The day after seeding, HEK293 cells were transfected with 1 μg of *CMV:mRFP-Q74* (ref. ^40^) together with *CMV:GFP-SPP* or *CMV:GFP* constructs. DNA was incubated at 80 °C for 5 min and mixed with FuGENE HD (Promega) in a 3:1 ratio (FuGENE:DNA) and 65 μl of Opti-MEM (Thermo Fisher Scientific) was added. The mixture was added to cells dropwise and cells were harvested for experiments after 72 h of incubation with refreshed DMEM. For microscopy, cells on coverslips were fixed with 4% paraformaldehyde and mounted for analysis with an Imager Z1 microscope (Zeiss).

### *C. elegans* strains and constructs

*C. elegans* was cultured on nematode growth medium seeded with *E. coli* (OP50) bacteria^66^. Worms were examined at the adulthood ages specified in the figure legends. For all the experiments, we used hermaphrodite worms. For motility assays, worms were transferred to M9 buffer. After 30 s of adaptation, body bends were counted for 30 s. A body bend was defined as a change in mid-body bend direction.

To construct the *SPP C. elegans* expression plasmid, pPD95.77 from the Fire Lab kit was digested with SphI and XmaI to insert 3.6 kb of the *sur5* promoter. The resultant vector was then digested with KpnI and EcoRI to excise GFP and insert a multi-cloning site containing KpnI, NheI, NotI, XbaI and EcoRI. SPP was PCR amplified from synthetic SPP and cloned into the vector with NheI and NotI sites (Supplementary Table 6). The construct was sequence verified.

AM716 (rmIs284[*F25B3.3p::Q67::YFP*]), AM101 (rmIs110[*F25B3.3p::Q40::YFP*]) and AM23 (rmIs298[*F25B3.3p::Q19::CFP*]) strains were provided by R. I. Morimoto^27^. For the generation of DVG343 (*rmIs284[F25B3.3p*::Q67::YFP], *ocbEx277*[*sur-5p::SPP*, *myo-3p::*GFP]) and DVG347 (rmIs110[*F25B3.3p::Q40::YFP*], *ocbEx279*[*sur-5p::SPP*, *myo-3p::*GFP]), a DNA mixture containing 50 ng μl^−1^ of the plasmids *sur5-p::SPP* and 20 ng μl^−1^ of pPD93_97 (*myo3-p::*GFP) was injected into the gonads of either adult AM716 or AM101 hermaphrodite animals using standard methods^67^. The corresponding control strains, DVG330 (*rmIs284[F25B3.3p*::Q67::YFP], *ocbEx165*[*myo-3p::GFP*]) and DVG346 (rmIs110[*F25B3.3p::Q40::YFP*],, *ocbEx278*[*myo-3p::GFP*]), were generated by microinjecting AM716 and AM101 worms with 20 ng μl^−1^ of pPD93_97.

### Filter trap and SDS–PAGE analysis

Plant tissues were lysed with native lysis buffer (300 mM NaCl, 100 mM Hepes, pH 7.4, 2 mM EDTA and 2% Triton X-100) supplemented with plant protease inhibitor (Merck). HEK293 cells were collected in nondenaturing lysis buffer (50 mM Hepes, pH 7.4, 150 mM NaCl, 1 mM EDTA and 1% Triton X-100) supplemented with EDTA-free protease inhibitor cocktail (Roche). Human cells were homogenized by passing 10× through a 27G needle. For filter trap analysis of *C. elegans*, we collected day p3 adult worms with M9 buffer. Worm extracts were obtained using glass-bead disruption in nondenaturing lysis buffer (50 mM Hepes, pH 7.4, 150 mM NaCl, 1 mM EDTA and 1% Triton X-100) supplemented with EDTA-free protease inhibitor cocktail. Cellular debris was removed by two to three centrifugation steps at 8,000*g* for 5 min at 4 °C. We collected the supernatants and measured protein concentration with Pierce BCA Protein Assay Kit (Thermo Fisher Scientific) and then 100 µg of protein extract was supplemented with SDS at a final concentration of 0.5%. The protein extract was loaded and filtered through a cellulose acetate membrane filter (GE Healthcare Life Sciences) in a slot blot apparatus (BioRad) coupled to a vacuum system. The membrane was washed with 0.2% SDS and protein aggregates were assessed by western blotting with either anti-GFP (AMSBIO, catalog no. TP401, 1:5,000), anti-polyQ (Merck, catalog no. MAB1574, clone 5TF1-1C2, 1:1,000) or anti-mCherry (Abcam, catalog no. ab167453, 1:5,000) as indicated in the corresponding figure legends. As secondary antibodies, we used IRDye 800CW donkey anti-mouse immunoglobulin (Ig)G (H + L; Licor, catalog no. 926-32212, 1:10,000) and RDye 800CW donkey anti-rabbit IgG (H + L; Licor, catalog no. 926-32213, 1:10,000). The extracts were also analyzed by SDS–PAGE/western blotting with anti-GFP (AMSBIO, catalog no. TP401, 1:5,000), anti-polyQ, anti-mCherry, anti-LC3 (Sigma-Aldrich, catalog no. L7543, 1:1,000), anti-β-actin (Abcam, catalog no. ab8226, clone mAbcam 8226, 1:5,000) and anti-α-tubulin (Sigma-Aldrich, catalog no. T6199, 1:5,000) as indicated in the figures. For western blotting, we used donkey anti-mouse horseradish peroxidase (HRP; Jackson ImmunoResearch, catalog no. 715-035-150, 1:10,000) and donkey anti-rabbit HRP (Jackson ImmunoResearch, catalog no. 711-035-152, 1:10,000) secondary antibodies.

### Western blotting analysis of plants

Plant material was ground in liquid N_2._ The powder was resuspended in ice-cold TKMES homogenization buffer (100 mM Tricine-potassium hydroxide, pH 7.5, 10 mM KCl, 1 mM MgCl2, 1 mM EDTA and 10% (w/v) sucrose) supplemented with 0.2% (v/v) Triton X-100, 1 mM DTT, 100 µg ml^−1^ of phenylmethylsulfonyl fluoride (PMSF), 3 µg ml^−1^ of E64 and plant protease inhibitor. After centrifugation at 10,000*g* for 10 min (4 °C), supernatant was collected for a second centrifugation. Protein concentration was determined with Pierce Coomassie Plus (Bradford) Protein Assay Kit. Total protein was SDS–PAGE separated, transferred to a nitrocellulose membrane and subjected to western blotting. The following antibodies were used for plant extracts: anti-GFP (AMSBIO, catalog no. TP401, 1:5,000), anti-plant actin (Agrisera, catalog no. AS132640, 1:5,000), anti-polyQ, anti-Hsp90-1 (Agrisera, catalog no. AS08346, 1:3,000), anti-Hsp70 (Agrisera, catalog no. AS08371, 1:3,000) and anti-ATG8 (Agrisera, catalog no. AS142769, 1:3,000).

### Proteasome activity

HEK293 cells were collected in proteasome activity assay buffer (50 mM Tris-HCl, pH 7.5, 10% glycerol, 5 mM MgCl_2_, 0.5 mM EDTA, 2 mM ATP and 1 mM DTT) and lysed by passing 10× through a 27G needle attached to a 1-ml syringe. Then, we centrifuged the samples (10,000*g*, 4 °C, 10 min) and collected the supernatants. Protein concentrations were determined using BCA Protein Assay Kit. To measure chymotrypsin-like proteasome activity, 25 μg of total protein was transferred to a 96-well microtiter plate (BD Falcon) and incubated with the fluorogenic proteasome substrate Z-Gly-Gly-Leu-AMC (Enzo). Fluorescence accumulation over time on degradation of the proteasome substrate (380-nm excitation, 460-nm emission) was measured with a microplate fluorometer (EnSpire, Perkin Elmer) every 5 min for 1 h at 37 °C.

### Interactome analysis

Q28 and Q69 seedlings age 7 d were lysed in lysis buffer (1% Triton X-100 and 50 mM Tris-HCl, pH 8.0) supplemented with 1× plant protease inhibitor cocktail and 25 mM *N*-ethylmaleimide. Samples were vortexed, centrifuged at 13,000*g* (10 min, 4 °C) and supernatants collected. HEK293 cells were lysed in modified radioimmunoprecipitation buffer (50 mM Tris-HCl, pH 7.4, 150 mM NaCl, 0.25% sodium deoxycholate (DOC), 1% IgPal, 1 mM PMSF and 1 mM EDTA) with protease inhibitor (Roche). Human cell lysates were centrifuged at 10,000*g* (10 min, 4 °C) and supernatants collected. For each sample, the same amount of total protein was incubated for 1 h with either anti-GFP antibody (1:500 for plants, 1:100 for HEK293) or negative control anti-IgG antibody (plants: Abcam, catalog no. ab46540, 1:500; HEK293: Cell Signaling, catalog no. 2729S, 1:100). Samples were then incubated with 50 μl of μMACS Micro Beads (Miltenyi) for 1 h at 4 °C, loaded on to pre-cleared μMACS column (catalog no. 130-042-701) and subjected to three washes using wash buffer 1 (50 mM Tris-HCl, pH 7.4, 150 mM NaCl, 5% glycerol and 0.05% Triton (plants) or 0.05% IgPal (HEK293)). Next, columns were washed 5× with wash buffer 2 (50 mM Tris-HCl, pH 7.4 and 150 mM NaCl). Columns underwent in-column tryptic digestion with 7.5 mM ammonium bicarbonate, 2 M urea, 1 mM DTT and 5 ng ml^−1^ of trypsin. Digested peptides were eluted using 50 μl of elution buffer 1 (2 M urea, 7.5 mM Ambic and 15 mM chloroacetamide) and incubated overnight at room temperature with shaking in the dark. The next day, samples were stage tipped for label-free quantification.

For plant sample data acquisition, we used a Q-Exactive Plus (Thermo Fisher Scientific) mass spectrometer coupled to an EASY nLC 1200 UPLC (Thermo Fisher Scientific), following the protocol detailed at https://www.ebi.ac.uk/pride/archive/projects/PXD041001. MS raw data were processed with MaxQuant (v.5.3.8)^68^ using default settings with label-free quantification (LFQ) enabled. MS2 spectra were searched against the *A. thaliana* UniProt database (UP6548, downloaded 26 August 2020), including a list of common contaminants. For HEK293 data acquisition, an Orbitrap Exploris 480 mass spectrometer (Thermo Fisher Scientific, granted by the German Research Foundation (DFG) under INST 1856/71-1 FUGG) equipped with FAIMSpro and coupled to a Vanquish neo (Thermo Fisher Scientific) was used, as detailed at https://www.ebi.ac.uk/pride/archive/projects/PXD044408. MS raw data were processed with MaxQuant (v.2.2) against a chimeric database of UniProt human reference database (UP5640, downloaded 4 January 2023) merged with SPP–GFP sequences, enabling the match-between-runs option between replicates. All downstream analyses were carried out on LFQ values with Perseus (plants: v.1.6.2.3; HEK293: v.1.6.15)^69^. Protein groups were filtered for potential contaminants and insecure identifications. The remaining IDs were filtered for data completeness in at least one group and missing values imputed by sigma downshift (0.3 σ width, 1.8 σ downshift).

### LiP–MS

Cells were lysed in LiP buffer (1 mM MgCl_2,_ 150 mM KCl and 100 mM Hepes, pH 7.4), homogenized by electro-douncer and centrifuged at 16,000*g* (10 min, 4 °C). Protein concentration was measured with the Pierce BCA Protein Assay Kit. Equal amounts of lysates were divided into PCR tube strips for LiP and control total level proteome analysis. The samples were incubated at 25 °C for 5 min. Subsequently, proteinase K (Sigma-Aldrich) was added to the LiP samples to a final concentration of 0.1 μg μl^−1^, incubated at 25 °C for 5 min and then incubated at 99 °C for 5 min. Finally, the samples were incubated at 4 °C for 5 min. The control samples without proteinase K were subjected to the same incubation procedure. After that, 10% DOC was added and samples were incubated on ice for 5 min. The samples were reduced using 5 mM DTT for 30 min at 37 °C, followed by alkylation with 20 mM iodoacetamide for 30 min. Then, we diluted the DOC concentration to 1% and added 1 μg of trypsin together with 0.1 μg of Lys-C to each sample, followed by overnight incubation at 37 °C. The enzymatic digestion was stopped by adding formic acid and the precipitated DOC was removed through filtration on 0.2-μm polyvinylidene difluoride (PVDF) membranes by spinning. Stage-tip extraction was used for cleaning up peptides.

Data acquisition was performed on an Orbitrap Exploris 480 mass spectrometer as detailed at https://www.ebi.ac.uk/pride/archive/projects/PXD044409. Raw measurements were aggregated to peptide and protein quantities by DIA-NN. Structural effects were calculated using the R package LiPAnalyzeR (https://github.com/beyergroup/LiPAnalyzeR). Differential expression of peptide and protein levels was calculated using linear models where the condition is the predictor and expression is the response variable. *P* values of structural and expression changes were adjusted using false discovery rate (FDR) correction. In addition to global effects, that is, within effect group correction, peptide-level effects were alternatively corrected per protein.

### Quantitative proteomics of *C. elegans*

Synchronized 3-day-old *C. elegans* adults were lysed in urea buffer (8 M urea, 2 M thiourea and 10 mM Hepes, pH 7.6) through glass-bead disruption. After this, the samples were cleared by centrifugation at 18,000*g* for 10 min. The supernatant was collected and protein concentration measured with the Pierce BCA Protein Assay Kit. The samples underwent a reduction process using 5 mM DTT for 1 h, followed by alkylation with 40 mM chloroacetamide for 30 min. Urea concentration was then reduced to 2 M and trypsin was added at a 1:100 (w/w) ratio for overnight digestion. The next day, samples were cleared by acidification and centrifugation at maximum speed for 5 min. Stage-tip extraction was employed for peptide cleanup.

Data acquisition was performed on an Orbitrap Exploris 480 mass spectrometer, as outlined in detail at https://www.ebi.ac.uk/pride/archive/projects/PXD044145. Then, samples were analyzed in DIA-NN v.1.8.1 (ref. ^70^). A UniProt *C. elegans* canonical database (UP1940, downloaded 4 January 2023) merged with the sequences of the Q67::YFP construct was used for library building. The DIA-NN output was further filtered based on library *q* value and global *q* value (≤0.01), along with a requirement of at least two unique peptides per protein, using R (4.1.3). LFQ values were computed using the DIA-NN R package (https://github.com/vdemichev/Diann-repackage)^70^. Subsequent analysis was carried out using Perseus 1.6.15 (ref. ^69^) by filtering for data completeness in at least one replicate group, followed by FDR-controlled Student’s *t*-tests. Gene Ontology Biological Process enrichment was performed with PANTHER Gene Ontology Resource (release 6 November 2023).

### Statistics and reproducibility

For quantifying filter traps, western blots and mRNA levels, data were presented as relative changes compared with the corresponding control conditions. To average independent experiments, we normalized test conditions to the corresponding control group measured at the same time in each replicate experiment. Accordingly, we performed statistical analysis of filter traps, western blots and mRNA levels by two-tailed Student’s *t*-tests for paired samples. For all other experiments, a two-tailed Student’s *t*-test for unpaired samples was utilized, because the control group data were compared with other conditions across different experiments without normalization for each individual experiment. GraphPad Prism (v.9.4.1) was employed for all statistical analyses, excluding proteomics. In proteomics experiments, significant differences between groups were assessed with Perseus (v.1.6.2.3 and v.1.6.15) using a two-sided Student’s *t*-test for unpaired samples. A permutation-based FDR approach was applied to correct for multiple testing.

No statistical methods were used to predetermine sample size, but our sample sizes are similar to those reported in previous publications using the same procedures^9,41,60^. Data distribution was assumed to be normal but this was not formally tested. After projection of LiP–MS samples on to a two-dimensional plane via principal component analysis, we detected that one LiP sample (proteinase K + trypsin) behaved similarly to other trypsin samples. Therefore, this sample was excluded from all analyses. In addition, a corresponding trypsin sample was excluded to calculate structural changes. No plants, worms or data points were excluded in other analyses. Plants, human cells and worms were distributed to the various groups of all experiments from single pulls. Data collection was not randomized. Data collection and analysis were not performed blind to the conditions of the experiments.

### Reporting summary

Further information on research design is available in the Nature Portfolio Reporting Summary linked to this article.

### Supplementary information

Reporting Summary

Supplementary Table 1 List of polyQ-containing proteins in *Arabidopsis thaliana.*

Supplementary Table 2 Analysis of proteomics data from co-immunoprecipitation experiments with anti-GFP (against citrine-Q28 or citrine-Q69) or negative control anti-IgG antibody in Q28- and Q69-expressing plants (*n* = 3 biological replicates, FDR-adjusted *P* value (*q*-value) <0.05 was considered significant).

Supplementary Table 3 Analysis of proteomics data from co-immunoprecipitation experiments with anti-GFP antibody comparing HEK293 cells expressing SPP–GFP with HEK293 cells expressing control GFP (*n* = 3, two-sided Student’s *t*-test, FDR < 0.05 was considered significant).

Supplementary Table 4 LiP–MS analysis comparing mRFP-Q74 HEK293 cells expressing SPP–GFP (*n* = 3 biological replicates) with mRFP-Q74 HEK293 cells expressing control GFP (*n* = 4; two-sided Student’s *t*-test, FDR < 0.05 was considered significant).

Supplementary Table 5 Shotgun label-free proteomics of total protein levels comparing 3-day-old adult Q67::YFP+SPP with control Q67::YFP worms (*n* = 4 biological replicates, FDR-adjusted *P* (*q* value) <0.05 was considered significant).

Supplementary Table 6 List of primers used in the present study.

### Source data

Source Data Fig. 1 Statistical source data.

Source Data Fig. 3 Statistical source data.

Source Data Fig. 4 Statistical source data.

Source Data Extended Data Fig. 1 Statistical source data.

Source Data Extended Data Fig. 2 Statistical source data.

Source Data Extended Data Fig. 3 Statistical source data.

Source Data Extended Data Fig. 6 Statistical source data.

Source Data Fig. 1 Unprocessed western blots.

Source Data Fig. 2 Unprocessed western blots.

Source Data Fig. 3 Unprocessed western blots.

Source Data Fig. 4 Unprocessed western blots.

Source Data Extended Data Fig. 1 Unprocessed western blots.

Source Data Extended Data Fig. 2 Unprocessed western blots.

Source Data Extended Data Fig. 5 Unprocessed western blots.

Source Data Extended Data Fig. 6 Unprocessed western blots.

## Data Availability

The authors declare that all data supporting the findings of the present study are available within the paper and its Supplementary Information files. Proteomics data have been deposited in the ProteomeXchange Consortium via the PRIDE partner repository with the dataset accession nos. PXD041001 (Q28 and Q69 interactome in plants), PXD044408 (SPP interactome in human cells), PXD044409 (LiP–MS in human cells) and PXD044145 (global protein levels in *C. elegans* on SPP expression). In proteomics experiments, MS2 spectra were searched against the canonical UniProt databases of *A. thaliana* (UP6548, downloaded 26 August 2020, https://www.uniprot.org/proteomes/UP000006548), *Homo sapiens* (UP5640, downloaded 4 January 2023, https://www.uniprot.org/proteomes/UP000005640) and *C. elegans* (UP1940, downloaded 4 January 2023, https://www.uniprot.org/proteomes/UP000001940).

## Code availability

Customized code used in this article (LiPAnalyzeR) can be accessed at https://github.com/beyergroup/LiPAnalyzeR.
