# Supplementary material for: In planta expression of human polyQ-expanded huntingtin fragment reveals mechanisms to prevent disease-related protein aggregation
Source: Nat Aging. 2023 Oct 2;3(11):1345–57. doi: 10.1038/s43587-023-00502-1 (PMC10645592; doi:10.1038/s43587-023-00502-1)
Supplement: Supplementary file 1 — Reporting Summary [file 43587_2023_502_MOESM1_ESM.pdf]

nature portfolio

Corresponding author(s): David Vilchez

Last updated by author(s): Aug 16, 2023

Reporting Summary

Nature Portfolio wishes to improve the reproducibility of the work that we publish. This form provides structure for consistency and transparency in reporting. For further information on Nature Portfolio policies, see our Editorial Policies and the Editorial Policy Checklist.

Statistics

For all statistical analyses, confirm that the following items are present in the figure legend, table legend, main text, or Methods section.

n/a	Confirmed	
☐	☒	The exact sample size (*n*) for each experimental group/condition, given as a discrete number and unit of measurement
☐	☒	A statement on whether measurements were taken from distinct samples or whether the same sample was measured repeatedly
☐	☒	The statistical test(s) used AND whether they are one- or two-sided<br>*Only common tests should be described solely by name; describe more complex techniques in the Methods section.*
☒	☐	A description of all covariates tested
☐	☒	A description of any assumptions or corrections, such as tests of normality and adjustment for multiple comparisons
☐	☒	A full description of the statistical parameters including central tendency (e.g. means) or other basic estimates (e.g. regression coefficient) AND variation (e.g. standard deviation) or associated estimates of uncertainty (e.g. confidence intervals)
☐	☒	For null hypothesis testing, the test statistic (e.g. *F*, *t*, *r*) with confidence intervals, effect sizes, degrees of freedom and *P* value noted<br>*Give P values as exact values whenever suitable.*
☒	☐	For Bayesian analysis, information on the choice of priors and Markov chain Monte Carlo settings
☒	☐	For hierarchical and complex designs, identification of the appropriate level for tests and full reporting of outcomes
☒	☐	Estimates of effect sizes (e.g. Cohen's *d*, Pearson's *r*), indicating how they were calculated

Our web collection on statistics for biologists contains articles on many of the points above.

Software and code

Policy information about availability of computer code

Data collection	No software was used
Data analysis	We used ImageJ (1.51s) to quantify densitometry of western blots. Annotated chloroplast proteins were analyzed for the presence of prion like domains (PrLDs) using the PLAAC software (Version 1: http://plaac.wi.mit.edu/). To identify intrinsically disordered regions (IDRs), we used the IUPred software (Version IUPred3: https://iupred.elte.hu/). To predict chloroplast transit peptides, we used ChloroP version1.1 (https://services.healthtech.dtu.dk/service.php?ChloroP-1.1). The photosynthetic activity of plants was analyzed using ImagingWin software (v.2.41a; Heinz Walz GmbH).<br><br>We used GraphPad Prism (version 9.4.1) for statistical analysis of all the data, with the exception of proteomics data.<br><br>For plant protein interactome experiments, mass spectrometric raw data were processed with MaxQuant (version 1.5.3.8) using default parameters. LFQ was performed using the LFQ mode and MaxQuant default settings. All downstream analyses were carried out on LFQ values with Perseus (versions 1.6.2.3). For human protein interactome experiments, mass spectrometric raw data were processed with Maxquant (version 2.2) using default parameters.<br><br>For C. elegans proteomics experiments, samples were analyzed in DIA-NN 1.8.1. LFQ values were calculated using the DIA-NN R-package from Demichev et al, Nat. Methods 2016 (code available in: https://github.com/vdemichev/Diann-repackage). Afterwards, analysis of results was performed in Perseus 1.6.15 by filtering for data completeness in at least one replicate group followed by FDR-controlled t-tests. Analysis of Gene Ontology Biological Process (GOBP) enrichment was performed with PANTHER Gene Ontology Resource (release 2023-06-11).<br><br>For analysis of LiP-MS, we used the R package LiPAnalyzeR, which can be accessed at https://github.com/beyergroup/LiPAnalyzeR

For manuscripts utilizing custom algorithms or software that are central to the research but not yet described in published literature, software must be made available to editors and reviewers. We strongly encourage code deposition in a community repository (e.g. GitHub). See the Nature Portfolio guidelines for submitting code & software for further information.

Data

Policy information about availability of data

All manuscripts must include a data availability statement. This statement should provide the following information, where applicable:

- Accession codes, unique identifiers, or web links for publicly available datasets
- A description of any restrictions on data availability
- For clinical datasets or third party data, please ensure that the statement adheres to our policy

The authors declare that all data supporting the findings of this study are available within the paper and its Supplementary Information files. Proteomics data have been deposited in the ProteomeXchange Consortium via the PRIDE partner repository with the data set identifiers PXD041001 (Q28 and Q69 interactome in plants), PXD044408 (SPP interactome in human cells), PXD044409 (LiP-MS in human cells), and PXD044145 (global protein levels in C. elegans upon SPP expression). In proteomics experiments, MS2 spectra were searched against the canonical Uniprot databases of A. thaliana (UP6548, downloaded 26/08/2020, https://www.uniprot.org/proteomes/UP000006548), h. sapiens (UP5640, downloaded 04.01.2023, https://www.uniprot.org/proteomes/UP000005640), and C. elegans (UP1940, downloaded 04/01/23, https://www.uniprot.org/proteomes/UP000001940).

Human research participants

Policy information about studies involving human research participants and Sex and Gender in Research.

Reporting on sex and gender	n/a
Population characteristics	n/a
Recruitment	n/a
Ethics oversight	n/a

Note that full information on the approval of the study protocol must also be provided in the manuscript.

Field-specific reporting

Please select the one below that is the best fit for your research. If you are not sure, read the appropriate sections before making your selection.

☒ Life sciences ☐ Behavioural & social sciences ☐ Ecological, evolutionary & environmental sciences

For a reference copy of the document with all sections, see nature.com/documents/nr-reporting-summary-flat.pdf

Life sciences study design

All studies must disclose on these points even when the disclosure is negative.

Sample size	No statistical methods were used to predetermine sample size. Exact sample sizes are indicated in the corresponding Figure legends and Supplementary Figure legends.<br>Sample sizes for filter traps, western blot, qPCR, and motility, were determined according to our previous laboratory experience and other studies using these assays (Koyuncu S et al, Nature 596:285-290 (2021), Llamas et al, Aging Cell 20: e13446; Lee HL et al; Nature Metabolism 1: 790-810 (2019); Koyuncu S et al, Nature Communications 9: 2886 (2018); Amrit FR et al, Methods 68: 465–475 (2014); Fatima A et al, Communications Biology 3: 262; Xin N et al, Journal of Cell Biology 221: e202201071 (2022), Segref A et al, Nature Communications 13: 5874 2022)).
Data exclusions	No data were excluded from the analyses.
Replication	At least three independent experiments for each assay were performed to verify the reproducibility of the findings (if there were two independent experiments, this is indicated in the figure legend). All the attempts of replication gave a similar outcome. Exact sample sizes/ number of independent experiments are indicated in the corresponding Figure legends, Supplementary Figure legends and Supplementary Data.
Randomization	For C. elegans experiments, worms were synchronized by picking young hermaphrodites adults and let them lay eggs for 6 hours. These young hermaphrodites were randomly picked from our maintenance plates. After egg laying for 6 hours, larvae were raised until adulthood and adult worms were then randomly allocated into the different experimental groups.

For experiment with human cell lines, cells with similar confluence were split and equal amounts of cells were transferred to new plates for experiments. The plates were randomly assigned to the different treatment conditions.
Plant, human cell and C. elegans samples were collected and lysed in random order. Data collection and analysis were not randomized

Blinding

The samples and different conditions were not processed in a blinded manner by the researchers participating in this study, but the critical experiments were repeated independently by at least 2 of the investigators involved in the study.
qPCR, filter trap and western blot experiments were not performed in a blinded manner as they rely on objective instrument measurements and/or provide indirect outputs. Data analysis of these experiments were not performed in a blinded manner as the investigators that performed the analysis also loaded the samples during the experiment and the corresponding outputs from measurement equipments were released in this order.
For experiments with direct outputs such as microscopy , the investigators were also not blinded when they analyzed the data as they would remember anyways the phenotype differences between conditions from when they collected the data. For these assays, the experiments and corresponding analysis were repeated independently .

Reporting for specific materials, systems and methods

We require information from authors about some types of materials, experimental systems and methods used in many studies. Here, indicate whether each material, system or method listed is relevant to your study. If you are not sure if a list item applies to your research, read the appropriate section before selecting a response.

Materials & experimental systems

n/a	Involved in the study
☐	☒ Antibodies
☐	☒ Eukaryotic cell lines
☒	☐ Palaeontology and archaeology
☐	☒ Animals and other organisms
☒	☐ Clinical data
☒	☐ Dual use research of concern

Methods

n/a	Involved in the study
☒	☐ ChIP-seq
☒	☐ Flow cytometry
☒	☐ MRI-based neuroimaging

Antibodies

Antibodies used

We used the following antibodies in this study:
For western blot:
anti-GFP (AMSBIO, TP401, 1:5,000). Polyclonal
anti-polyQ (Merck, MAB1574, 1:1000). Monoclonal, clone number: 5TF1-1C2
anti-mCherry (Abcam, ab167453, 1:5,000). Polyclonal
anti-Actin (Agrisera, AS132640, 1:5,000). Polyclonal
anti-β-actin (Abcam, ab8226, 1:5,000). Monoclonal, clone number: mAbcam 8226
anti-Hsp90-1 (Agrisera, AS08346, 1:3,000). Polyclonal
anti-Hsp70 (Agrisera, AS08371, 1:3,000). Polyclonal
anti-ATG8 (Agrisera, AS142769, 1:1,000). Polyclonal.
anti-α-tubulin (Sigma, T6199, 1:5,000). Monoclonal, clone number: DM1A
anti-LC3 (Sigma, L7543, 1:1,000). Polyclonal
Donkey Anti-Mouse HRP (Jackson ImmunoResearch, 715-035-150, 1:10,000). Polyclonal.
Donkey Anti-Rabbit HRP (Jackson ImmunoResearch, 711-035-152, 1:10,000). Polyclonal.

For filter traps:
anti-GFP (AMSBIO, TP401, 1:5,000). Polyclonal
anti-mCherry (Abcam, ab167453, 1:5,000). Polyclonal
anti-polyQ (Merck, MAB1574, 1:1000). Monoclonal, clone number: 5TF1-1C2
IRDye 800CW Donkey anti-Rabbit IgG (H + L) (Licor, 926-32213, 1:10,000). Polyclonal.
IRDye 800CW Donkey Anti-Mouse IgG (H+L) (Licor, 926-32212, 1:10,000). Polyclonal.

Validation

Validation of antibodies were done by the stated manufacturer's, this study, or previous publications and supported by the publications indicated in the manufacturer's website.

*anti-GFP (AMSBIO, TP401). This antibody has been validated for filter trap and western blot in C. elegans and human cells in our previous publications: PMID: 27892468; PMID: 30038412; PMID: 34172445; PMID: 34321666
* anti-polyQ (Merck, MAB1574, clone 5TF1-1C2, 1:1000). This antibody has been validated for filter trap and western blot in C. elegans and human cells in our previous publications: PMID: 30038412; PMID: 34172445; PMID: 37118550
* anti-mCherry (Abcam, ab167453, 1:5,000). According to manufacturer's web page ab167453 https://www.abcam.com/mcherry-antibody-ab167453.html
* anti-Actin (Agrisera, AS132640, 1:5,000). Validation and references available at: https://www.agrisera.com/en/artiklar/act-actin.html
*anti-β-actin (Abcam, ab8226, clone mAbcam 8226, 1:5,000) was used according to the manufacturer's instructions and our previous publications: PMID: 27892468; PMID: 30038412; PMID: 32451438
*anti-Hsp90-1 (Agrisera, AS08346, 1:3,000). Validation and references at: https://www.agrisera.com/en/artiklar/hsp90-heat-shock-

protein-90.html
* anti-Hsp70 (Agrisera, AS08371, 1:3,000). anti-Hsp70 [1:3000]. Validation and references at: https://www.agrisera.com/en/artiklar/hsp70-heat-shock-protein-70-cytoplasmic.html
* anti-ATG8 (Agrisera, AS142769, 1:1,000). Validation and references at: https://www.agrisera.com/en/artiklar/atg8.html
*anti-α-tubulin (Sigma, T6199, 1:5,000). This antibody was validated as a loading control for western blot analysis in C. elegans in our previous publications: PMID: 32451438; PMID: 27892468; PMID: 34172445; PMID: 34321666; PMID: 37118550
* anti-LC3 (Sigma, L7543, 1:1,000). Validation and references: https://www.sigmaaldrich.com/DE/en/product/sigma/l7543. PMID: 30038412
* Anti-Mouse HRP (Jackson ImmunoResearch, 715-035-150, 1:10,000). Validation and references: https://www.jacksonimmuno.com/catalog/products/715-035-150
* Anti-Rabbit HRP (Jackson ImmunoResearch, 711-035-152, 1:10,000). Validation and references: https://www.jacksonimmuno.com/catalog/products/711-035-152
* IRDye 800CW Donkey anti-Rabbit IgG (H + L) (Licor, 926-32213, 1:10,000). Validation and references: https://www.licor.com/bio/reagents/irdye-800cw-donkey-anti-rabbit-igg-secondary-antibody
* IRDye 800CW Donkey Anti-Mouse IgG (H+L) (Licor, 926-32212, 1:10,000). Validation and references: https://www.licor.com/bio/reagents/irdye-800CW-donkey-anti-mouse-igg-secondary-antibody

Eukaryotic cell lines

Policy information about cell lines and Sex and Gender in Research

Cell line source(s)	In this study, we used the human HEK293 cell line (HEK293T/17) obtained from the American Type Culture Collection (ATCC). Catalog number: CRL-11268.
Authentication	The HEK293 (HEK293T/17) cell line commercially obtained from ATTC has not been authenticated in our laboratory.
Mycoplasma contamination	The HEK293 (HEK293T/17) cell line used in this study was tested for mycoplasma contamination at least once every 3 weeks. No mycoplasma contamination was detected.
Commonly misidentified lines (See ICLAC register)	The HEK293 (HEK293T/17) cell line used in this paper is not listed in the database of commonly misidentified cell lines maintained by ICLAC (version 12, released 16th January 2023)

Animals and other research organisms

Policy information about studies involving animals; ARRIVE guidelines recommended for reporting animal research, and Sex and Gender in Research

Laboratory animals	In this study, we used different Caenorhabditis elegans strains. For all the experiments, we used hermaphrodites worms.<br><br>The age of the worms is indicated in the corresponding figure legends. For most of the experiments, we analyzed day 3-adult worms. In Figure 4m, we analyzed worms at day 1, 3, 5, and 7 of adulthood.<br><br>The C. elegans strains used in this study were:<br>AM716 (rmIs284[F25B3.3p::Q67::YFP])<br>DVG343 (N2, ocbEx277[sur-5p::SPP, myo-3p::GFP])<br>DVG330 (rmIs284[F25B3.3p::Q67::YFP], ocbEx165[myo-3p::GFP])<br>AM23 (rmIs298[F25B3.3p::Q19::CFP])<br>AM101 (rmIs110[F25B3.3p::Q40::YFP])<br>DVG346 (rmIs110[F25B3.3p::Q40::YFP],, ocbEx278[myo-3p::GFP])<br>DVG347 (rmIs110[F25B3.3p::Q40::YFP], ocbEx279[sur-5p::SPP, myo-3p::GFP])<br><br>All the Arabidopsis thaliana lines used in this work are in Columbia-0 (Col-0) ecotype. WT, toc159 (Woodson, J. D. et al. Science 350, 450-454, (2015) and Ling, Q. et al. Science 363, (2019)), and cct8-2 (Llamas, E. et al. Aging Cell 20, e13446, (2021)) were used in this study. The age of the plants is indicated in the corresponding figure legends.
Wild animals	The study did not involve wild animals
Reporting on sex	The study did not involve samples collected from the field.
Field-collected samples	No field collected samples were used in the study.
Ethics oversight	According to the “Zentrale Kommission für die Biologische Sicherheit” (ZKBS), the responsible entity inside the Bundesamt für Verbraucherschutz und Lebensmittelsicherheit to assess the risk of Genetically Modified Organisms (GMO), genetic work with C. elegans is classified as risk group 1 (biological safety level 1: S1). Accordingly, we performed work on C. elegans in a S1-laboratory. The use of GMO in Germany is regulated by the “Gentechnik-Gesetz”, and we followed the guidelines applying to S1 work with GMO (i.e., documentation of the project and of the, exact description of the creation and maintenance of the genetic modification or correct waste treatment).

Note that full information on the approval of the study protocol must also be provided in the manuscript.
