# Supplementary figures and images for: In planta expression of human polyQ-expanded huntingtin fragment reveals mechanisms to prevent disease-related protein aggregation
Source: Nat Aging. 2023 Oct 2;3(11):1345–57. doi: 10.1038/s43587-023-00502-1 (PMC10645592; doi:10.1038/s43587-023-00502-1)

Fig. 1i

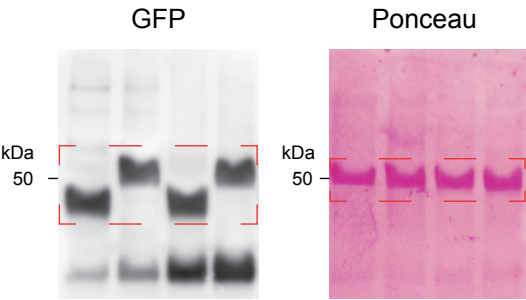

Supplement: Supplementary file 15 — Unprocessed western blots. [file 43587_2023_502_MOESM15_ESM.pdf]

**Fig. 2d**

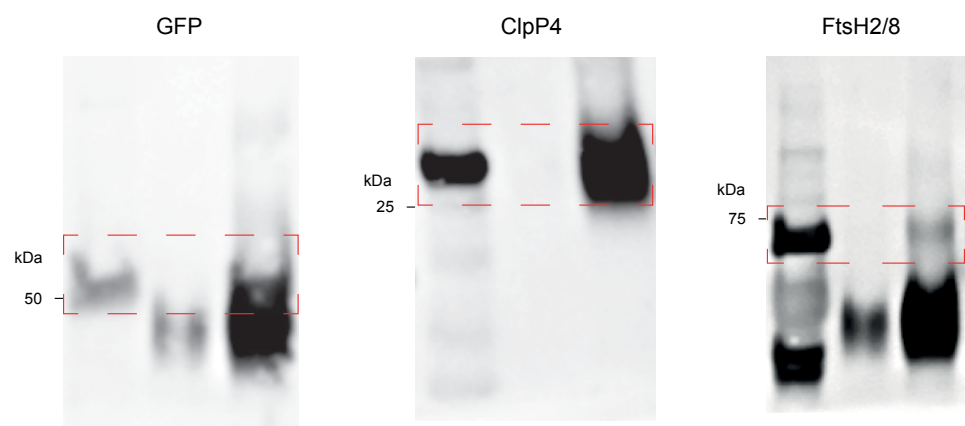

Supplement: Supplementary file 16 — Unprocessed western blots. [file 43587_2023_502_MOESM16_ESM.pdf]

**Fig. 3c**

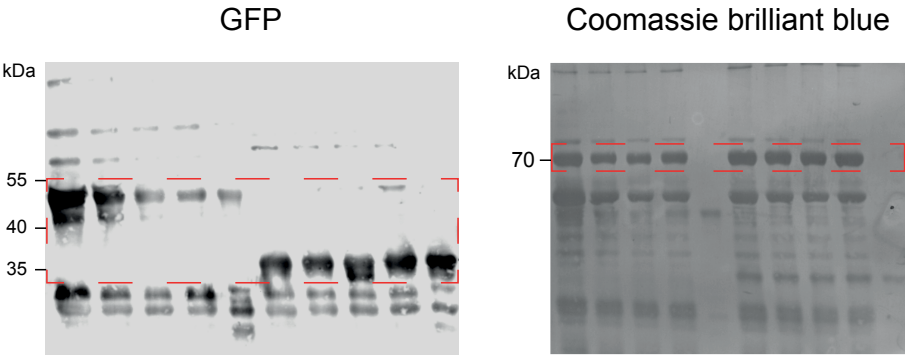

**Fig. 3d**

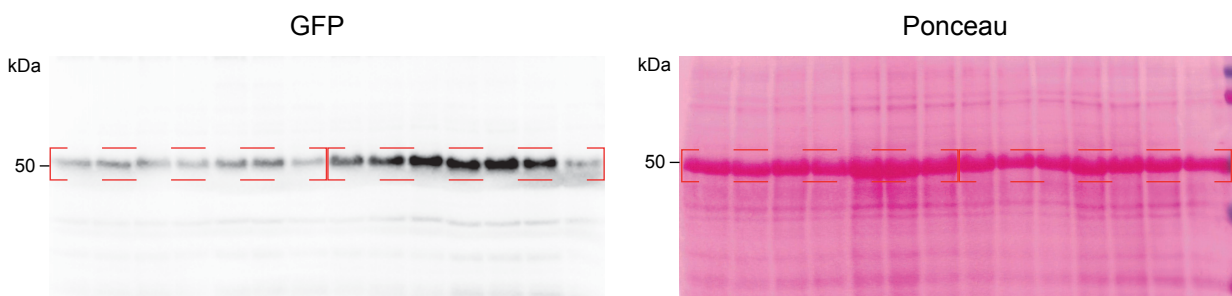

**Fig. 3i**

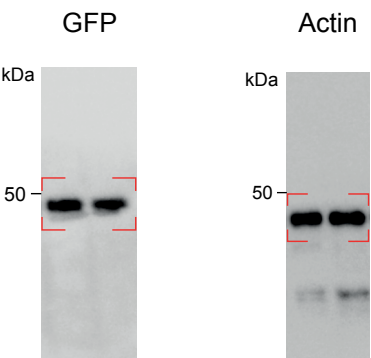

**Fig. 3k**

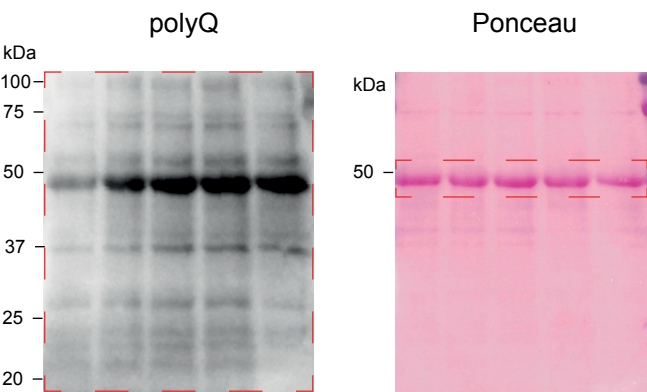

Supplement: Supplementary file 17 — Unprocessed western blots. [file 43587_2023_502_MOESM17_ESM.pdf]

**Fig. 4e**

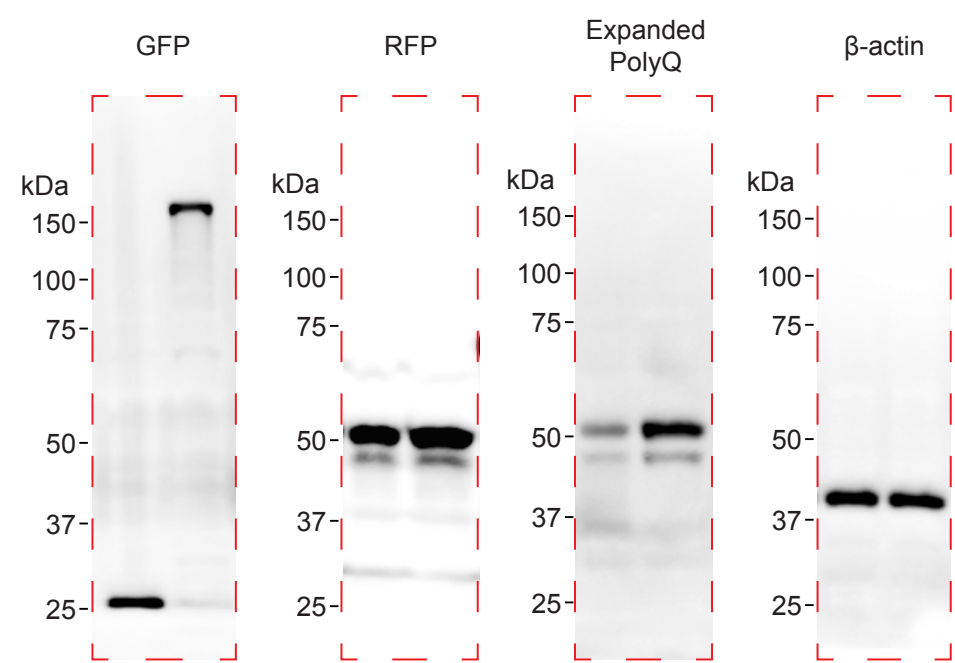

**Fig. 4j**

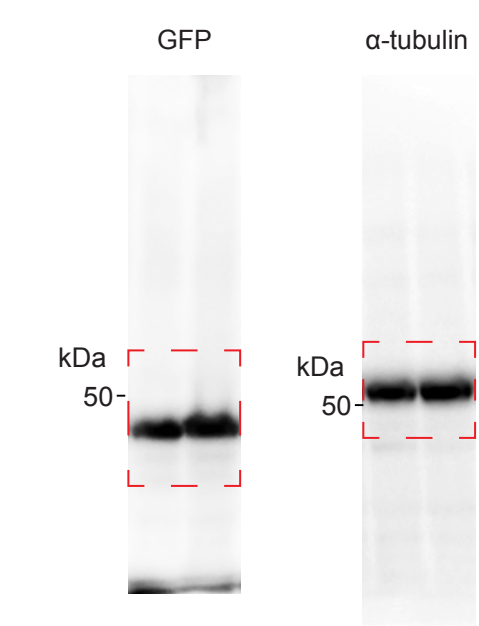

Supplement: Supplementary file 18 — Unprocessed western blots. [file 43587_2023_502_MOESM18_ESM.pdf]

Extended Data Fig. 1a

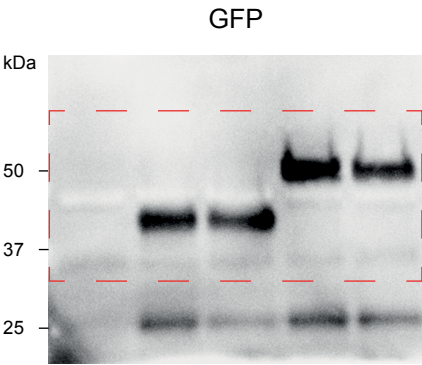

Extended Data Fig. 1g

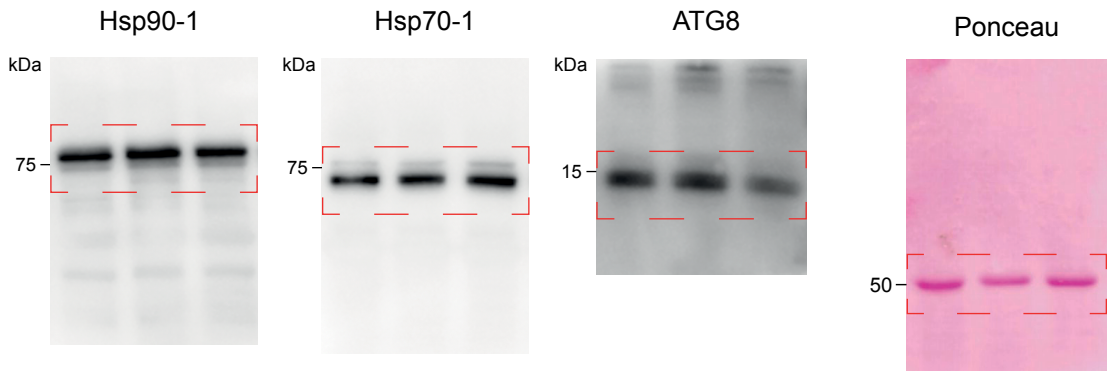

Supplement: Supplementary file 19 — Unprocessed western blots. [file 43587_2023_502_MOESM19_ESM.pdf]

Extended Data Fig. 2a

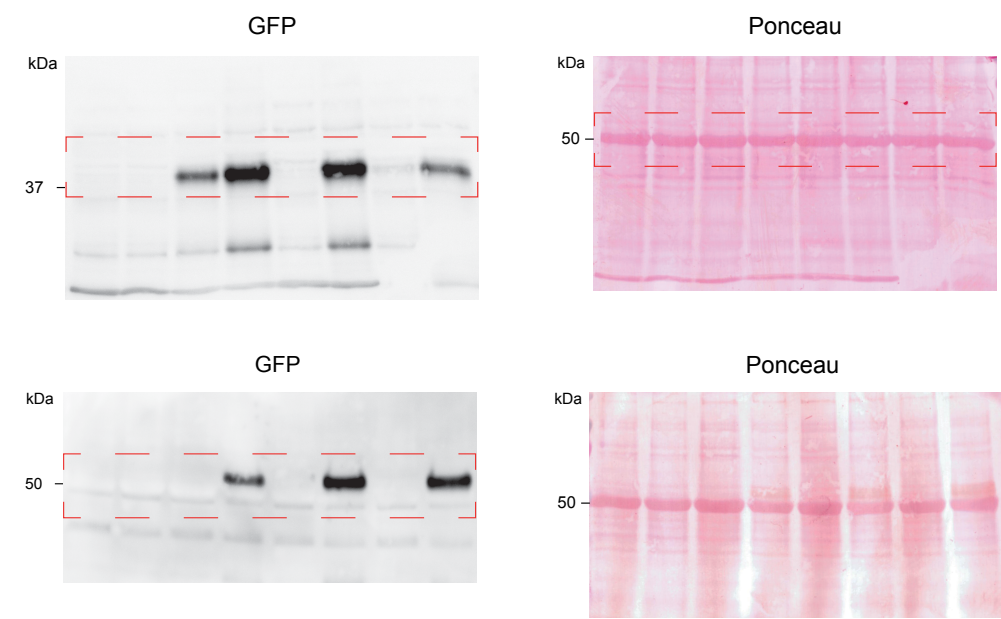

Supplement: Supplementary file 20 — Unprocessed western blots. [file 43587_2023_502_MOESM20_ESM.pdf]

Extended Data Fig. 5c

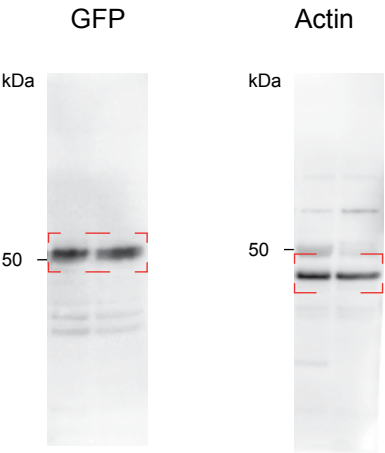

Extended Data Fig. 5f

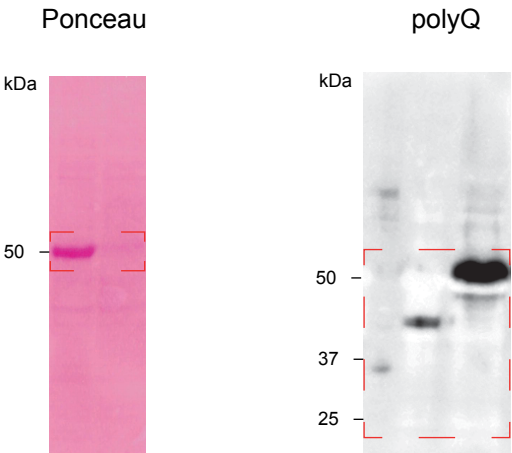

Supplement: Supplementary file 21 — Unprocessed western blots. [file 43587_2023_502_MOESM21_ESM.pdf]

Extended Data Fig. 6a

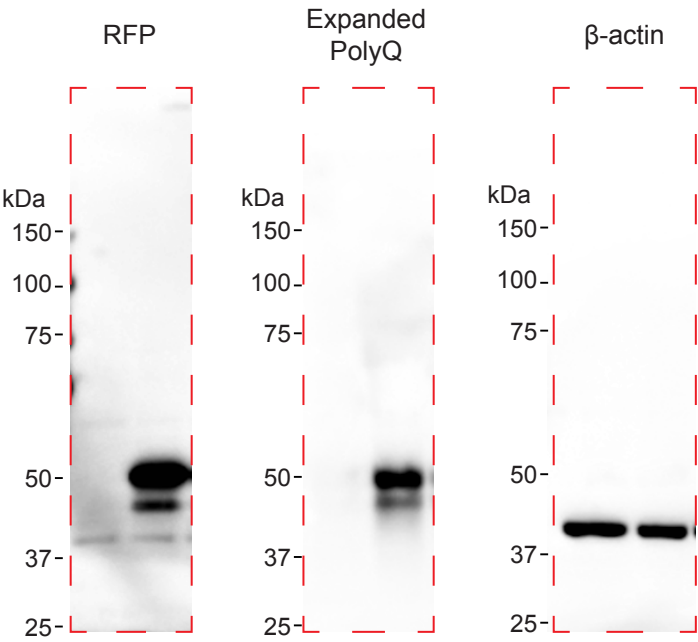

Extended Data Fig. 6d

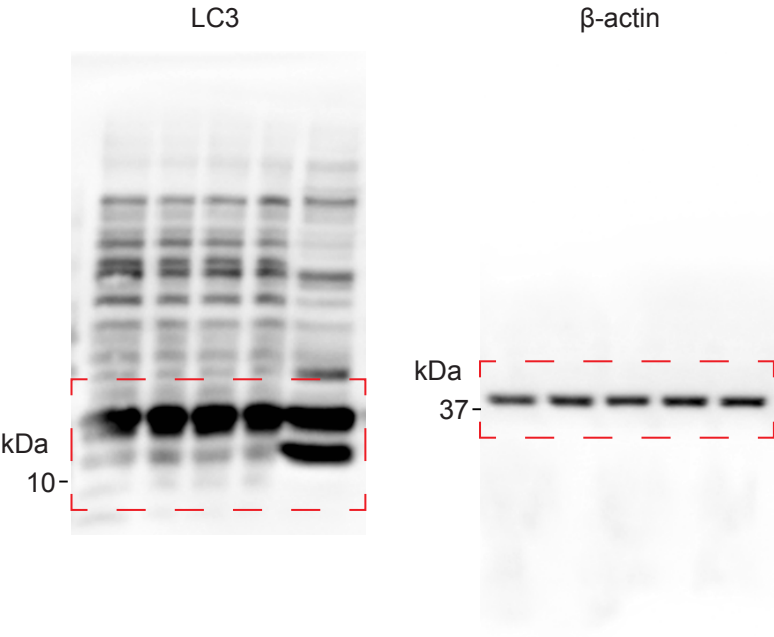

Supplement: Supplementary file 22 — Unprocessed western blots. [file 43587_2023_502_MOESM22_ESM.pdf]
